# Exosome-delivered and Y RNA-derived small RNA suppresses influenza virus replication

**DOI:** 10.1186/s12929-019-0553-6

**Published:** 2019-08-15

**Authors:** Yuag-Meng Liu, Chung-Hsin Tseng, Yi-Chun Chen, Wen-Ya Yu, Meng-Yen Ho, Chia-Yin Ho, Michael M. C. Lai, Wen-Chi Su

**Affiliations:** 1 Graduate Institute of Clinical Medical Science, China Medical University, No.91, Xueshi Rd., North Dist, Taichung City, 404 Taiwan; 2 Division of Infectious Diseases, Changhua Christian Medical Foundation Changhua Christian Hospital, No. 135, Nanxiao St., Changhua City, Changhua County 500 Taiwan; 3 Department of Microbiology and Immunology, College of Medicine, National Cheng Kung University, No. 1, Daxue Rd., East Dist, Tainan City, 701 Taiwan; 4 Center of Infectious Disease and Signaling Research, National Cheng Kung University, No. 1, Daxue Rd., East Dist, Tainan City, 701 Taiwan; 5 Research Center for Emerging Viruses, China Medical University Hospital, No.91, Xueshi Rd., North Dist, Taichung City, 404 Taiwan; 6 Graduate Institute of Biomedical Sciences, China Medical University, No.91, Xueshi Rd., North Dist, Taichung City, 404 Taiwan; 7 Institute of Molecular Biology, Academia Sinica, No. 128, Sec. 2, Academia Rd., Nangang Dist, Taipei City, 115 Taiwan

**Keywords:** Influenza virus, Y RNA, Hsa-miR-1975, Interferon, Exosome

## Abstract

**Background:**

Multiple interplays between viral and host factors are involved in influenza virus replication and pathogenesis. Several small RNAs have recently emerged as important regulators of host response to viral infections. The aim of this study was to characterize the functional role of hsa-miR-1975, a Y5 RNA-derived small RNA, in defending influenza virus and delineate the mechanisms.

**Methods:**

We performed high throughput sequencing of small RNAs in influenza virus-infected cells to identify up- or down- regulated small RNA species. The expression of the most abundant RNA species (hsa-miR-1975) was validated by stem-loop reverse transcription-polymerase chain reaction (RT-PCR). Antiviral effects of hsa-miR-1975 were confirmed by Western Blot, RT-PCR and plaque assay. In vitro perturbation of hsa-miR-1975 combined with exosomes isolation was used to elucidate the role and mechanism of hsa-miR-1975 in the context of antiviral immunity.

**Results:**

Small RNA sequencing revealed that hsa-miR-1975 was the most up-regulated small RNA in influenza virus-infected cells. The amount of intracellular hsa-miR-1975 increased in the late stage of the influenza virus replication cycle. The increased hsa-miR-1975 was at least partially derived from degradation of Y5RNA as a result of cellular apoptosis. Unexpectedly, hsa-miR-1975 mimics inhibited influenza virus replication while hsa-miR-1975 sponges enhanced the virus replication. Moreover, hsa-miR-1975 was secreted in exosomes and taken up by the neighboring cells to induce interferon expression.

**Conclusions:**

Our findings unravel a critical role of Y-class small RNA in host’s defense against influenza virus infection and reveal its antiviral mechanism through exosome delivery. This may provide a new candidate for targeting influenza virus.

**Electronic supplementary material:**

The online version of this article (10.1186/s12929-019-0553-6) contains supplementary material, which is available to authorized users.

## Introduction

Influenza virus continually threatens public health and antiviral drug resistance has become a major concern for clinical management [1]. Accordingly, identification of host factors involved in viral replication may aid in understanding the interplay between virus and host, as well as in finding new targets for the development of antiviral compounds. Current evidence indicates that host microRNAs (miRNA, typically 21–25 nucleotides long) can play significant roles in the host-virus interaction. Some small RNAs facilitate influenza A virus (IAV) replication. For example, miR-141 suppresses transforming growth factor (TGF)-β2 and miR-9 targets monocyte chemoattractant protein 1-induced protein 1 (MCPIP1) to benefit IAV life cycle [2, 3]. On the other hand, several small RNAs participate in the host defensive response to IAV [4–8]. Possible mechanisms of how these small RNAs regulate cellular antiviral response were proposed. For instance, miR-483-3p targets *RNF5* and *CD81*, which are regulators of RIG-I pathways [9]. Their downstream signaling augments the expression of IFN-β upon influenza virus infection [9].

Y RNAs, typically 83–112-nucleotides long, belong to another class of small non-coding RNAs and are involved in a range of cellular processes, including DNA replication, RNA stability and cellular stress responses [10]. Recent studies indicate that Y RNAs can be degraded into small fragments, called Y RNA-derived small RNAs (YsRNAs). The nature and functions of YsRNAs are currently unknown, and, thus, they have been removed from miRBase, which is the primary database for miRNAs [11]. It is speculated that YsRNAs are further processed into microRNA-like small RNAs but its definite signal pathway had not been clearly elucidated [12].

Extracellular vesicles, including microvesicles and exosomes, contain mRNA, miRNA and other small noncoding RNA species [13–16]. Of the different extracellular vesicles, exosomes have been the most studied in the context of infection. RNA present within exosomes is biologically active, implying that the RNA can modulate the protein profile and cellular state of the recipient cell [17]. Functions and applications of exosomally transferred RNA include promoting or inhibiting tumor progression, predicting drug response in cancer treatment, mediating cross-talk between the feto-placental unit and the mother during pregnancy and modulating inflammatory response [9, 18–24]. However, cellular origin and the pathological status of cell will determine the content encapsulated within exosomes. This phenomenon pointed to the fact that packaging of RNA into exosomes is an intricately regulated event [21, 25, 26].

By sequencing small RNAs from cells with and without IAV infection, we identified hsa-miR-1975, one of the Y5 RNA-derived small RNAs, as the most up-regulated small RNA after influenza virus infection. Furthermore, we demonstrated that a synthetic hsa-miR-1975 mimic inhibited influenza virus replication through the production of interferon. Finally, we revealed the mechanism by which hsa-miR-1975 exerted its antiviral effect.

## Materials and methods

### Cell cultures, reagents, and viruses

Human lung adenocarcinoma epithelial A549 cells were obtained from the Bioresource Collection and Research Centre (BCRC, Taiwan). Madin-Darby canine kidney (MDCK) cells were obtained from ATCC. We cultured A549 cells in F-12 K medium (Invitrogen) by adding 10% FBS (Thermo Scientific), 100 U/mL penicillin G and 100 μg/mL streptomycin. We cultured MDCK cells in DMEM (Invitrogen) by adding 10% FBS (Thermo Scientific), 100 U/mL penicillin G, and 100 μg/mL streptomycin. We maintained cells in a humidified incubator at 37 °C with 5% CO2. The A/WSN/33 (H1N1) (WSN) strain of influenza A virus (IAV) was mainly used in the studies. NC99 (H1N1) and W10 (H3N2) were also used to see whether the expression of small non-coding RNA presented after infection with different viral strains.

### Small RNA deep sequencing

A549 cells were infected with A/WSN/33 virus (MOI = 5) for 25 h and harvested for RNA extraction. RNA from uninfected A549 cells was used as a control. The total RNAs were subjected to gel electrophoresis and the RNA bands corresponding to size fractions of 18–40 nucleotide were isolated and extracted for small RNA sequencing. Small-RNA library construction and deep sequencing were done by Welgene Biotech (Taipei, Taiwan). Quality control of RNA revealed the following: the ratios of A230/260 were 2.67 and 1.84, respectively, for WSN infection and without WSN infection. The ratio of A260/280 was 1.96 for both. The ratio of 28S/18S was also 2 for both.

Samples were prepared by using an Illumina sample-preparation kit. In brief, total RNA was ligated with 3′ and 5′ adaptors and reverse-transcribed into cDNA. Polymerase chain reaction amplification was performed to amplify cDNA. cDNA constructs were fractionated by size and purified by using polyacrylamide gel electrophoresis. The libraries were sequenced on an Illumina instrument. Illumina software was used to analyze the sequencing data. Here, 12,057,156 and 11,067,109 reads were obtained for virus-infected and uninfected samples, respectively.

### Quantitative RT-PCR

We isolated cellular RNA by using a High Pure RNA Isolation Kit (Roche Diagnostics) according to the manufacturer’s protocol. We created cDNA by using the SuperScript III First-Strand Synthesis System (Invitrogen). The cDNA of YsRNA was prepared by using stem-loop reverse transcription. The standard TaqMan method using the Universal Probe Library System (Roche Diagnostics) was employed for real-time PCR analysis. GAPDH was used as a control for the normalization of cellular RNA and intracellular viral RNA. U24 RNA was used as a control for normalization of human small RNA.

### Primers for quantitative RT-PCR

The primers for reverse transcription are oligo (dT) 20 and IAV-specific RT primer (uni-12; 5′-AGCAAAAGCAGG-3′). The primers and probes are the following: for IAV_NP segment: sense 5′-GATGGAGACTGATGGAGAACG-3’and antisense 5′-TCATTTTTCCGACAGATGCTC-3′ with Universal Probe 59; GAPDH: sense 5′-AGCCACATCGCTCAGACAC-3′ and antisense 5′-GCCCAATACGACCAAATCC-3′ with Universal Probe 60; hsa-miR-1975: RT stem-loop primer: 5′-GTTGGCTCTGGTGCAGGGTCCGAGGTATTCGCACCAGAGCCAACAGCTAG-3′; qPCR primers: Forward 5′-CCCCCACAACCGCGC-3′ and Reverse 5′-GTGCAGGGTCCGAGGT-3′ with Universal Probe 21; hsa-Y5: RT stem-loop primer: 5′-TTGGCTCTGGTGCAGGGTCCGAGGTATTCGCACCAGAGCCAACAAAACAG; qPCR primers: Forward 5′-GTCCGAGTGTTGT- GGGTTATTG-3′ and Reverse 5′-GTGCAG- GGTCCGAGGT-3′ with Universal Probe 21; U24: RT stem-loop primer: GTTGGCTCTGGTGCAGGGTCCGAGGTATTCGCACCAGAGCCAACTGCATCA; qPCR primers: Forward 5′-TTGCTATCTGAGAGATGGTGATGAC-3′ and Reverse 5′-GTGCAGGGTCCGAGGT-3′ with Universal Probe 21; interferon B: Forward 5′-CTTTGCTATTTTCAGACAAGATTCA-3′ and Reverse 5′-GCCAGGAGGTTCTC AACAAT-3′ with Universal Probe 20.

### Quantification of miR-1975 and Y5

Serial dilution of hsa-miR-1975 mimic (purchased from MDBio, Taiwan) and human Y5 mimic (purchased from Dharmacon) were prepared. 5 different amounts of hsa-miR-1975 mimic and human Y5 mimic were used for RT-PCR. The amount of RNA mimics and Ct values were used to construct standard equations. We applied the equations to calculate the amount of hsa-miR-1975 and Y5 in cells.

### Western blotting analysis

The antibodies for NP, GAPDH, CD63, CD81, TSG101 and HSP70 were purchased from Genetex (no. GTX629633, GTX100118, GTX17441, GTX31831, GTX10255 and GTX11573). The antibodies for HA and calnexin were purchased from Millipore (no. 04–902 and AB2301). Cell lysates were prepared using M-PER mammalian protein extraction reagent (Thermo Scientific) with additional protease inhibitors, subjected to electrophoresis on a SDS-PAGE, and transferred onto a Hybond-P membrane. The membrane was probed with the indicated primary and appropriate secondary antibodies, detected using an enhanced chemiluminescence detection kit, and then imaged by Image-Quant LAS4000.

### Y5 mimic, miR-1975 mimic and miR-1975 scramble

Y5 mimic and miR-1975 mimic were purchased from Dharmacon. Scrambled miR-1975 RNA was purchased from MDBio, Taiwan. hsa-miR-1975 mimic, which sequence is identical to hsa-miR-1975, and Scrambled miR-1975 sequences are as follows:

Y5 mimic (5′-AGUUGGUCCGAGUGUUGUGGGUUAUUGUUAAGUUGAUU-UAACAUUGUCUC CCCCCACAACCGCGCUUGACUAGCU-3′) miR-1975 mimic (5′- CCCCCACAACCGCGCUUGACUAGCU-3′) and miR-1975 scramble (5′ –AUAGGCUCCGACGCUCCACACCCUC-3′).

### Transfection

A549 or Vero cells were transfected with miR-1975 mimic, control SiRNA or miR-1975 scramble by using a commercial liposome reagent, DMRIE-C (Invitrogen), according to the protocol provided by the manufacturer. After transfection for 48 h, cells were collected for further analysis or infected with WSN virus.

### Plaque assay

We infected MDCK cells with serial 10-fold dilutions of IAV for 1 h. We washed them twice with PBS, and then overlaid them with 0.5% agarose-containing MEM-alpha medium. We fixed the cells with 10% formaldehyde and stained cells with 0.1% crystal violet solution 2 days later for counting colony-forming units (CFUs).

### Construction of hsa-miR-1975 sponge

Plasmid carrying hsa-miR-1975 sponge was constructed and sequenced at National RNAi Core Facility, Academia Sinica (Taipei, Taiwan). In short, 11 repeats of sponge sequence 5′-AGCTAGTCAAGCGAAATGTGGGGG-3′ were inserted into 3′ of GFP cDNA. Each sponge sequence is separated by spacer sequence CTAC. The sponge sequence is mostly complementary to hsa-miR-1975 sequence.

### Exosomes isolation, purification, characterization and RNases treatment

We added ExoQuick-TC (System Biosciences) to the clarified cell culture medium at 1:5 ratio (by volume) to precipitate exosomes. The tube containing the mixture was inverted several times and then incubated overnight at 4 °C. The next day, the sample was centrifuged twice at 1500 *g* for 30 and 5 min, respectively, in order to remove the supernatant. The pellet was resuspended in 100 μL of PBS for Western Blot of exosome marker, quantitative RT-PCR and treating recipient cells. Western Blot of CD63, CD81, TSG101, HSP70, and calnexin of cellular and exosomal specimens were performed to characterize exosomes. We also diluted exosome pellets in 500 μL PBS. Size of exosomes were determined by using the dynamic light scattering technique (Zetasizer Nano ZS, Malvern Instruments, UK).

To confirm RNA transcripts are contained within the exosomes, RNase was applied when preparation of the exosomes to remove outside contaminants of RNA. Exosomes pellets were diluted in 50 μL PBS and treated with Ambion RNase cocktail 2.5 μL at 37 °C for 15 min.

### Poly(I:C) treatment

Polyinosinic:polycytidylic acid potassium salt (PolyI:C) was suspended at 1 μg/ml in nuclease-free water and transfected into cells. At 5 h post-transfection, the cultured media was replaced with fresh media. Cells were harvested for RNA extraction at 24 h post transfection.

### Exosomes isolation from donor cells, quantification of exosomes and recipient cells treatment

2 × 10^6^ A549 cells were grown on 3 plates (10-cm dish) and incubated overnight. Two plates of cells were transfected with control SiRNA and one plate of cells was transfected with miR-1975 inhibitor on the second day. We treated 2 plates of cells, one was transfected with control SiRNA and the other was transfected with miR-1975 inhibitor, with Poly(I:C) on third day. We collected cell supernatant on fourth day. We added Exo-Quick to the cell supernatant and incubated at 4 °C overnight. Exosome pellets were diluted in 600 μL PBS and stored in − 80 °C. Protein concentrations of exosomes were measured by Bradford protein assay. We treated 2 × 10^5^ recipient A549 cells with 100 μL of the exosomes and infected cells with WSN 24 h after exosomes treatment.

### Cell survival assay

Cell survival was assessed by the MTS assay. The A549 cells were seeded onto 96-well plates and then transfected with mock, control SiRNA, hsa-miR-1975 mimic, hsa-miR-1975 inhibitor at concentrtion of 200 nM for 48 h. For virus infection group, A549 cells were infected with WSN at MOI = 1 for 24 h.

### Statistics

Quantitative variables were compared by one-Way ANOVA or Student’s *t*-test as appropriate. For all analyses, a *P* value below 0.05 was considered statistically significant for two-tailed tests. The SPSS (SPSS Inc., Chicago, IL, USA) was used for statistical analysis.

## Results

### Identification of human small RNA species involved in influenza a virus infection

**Fig. 1:**
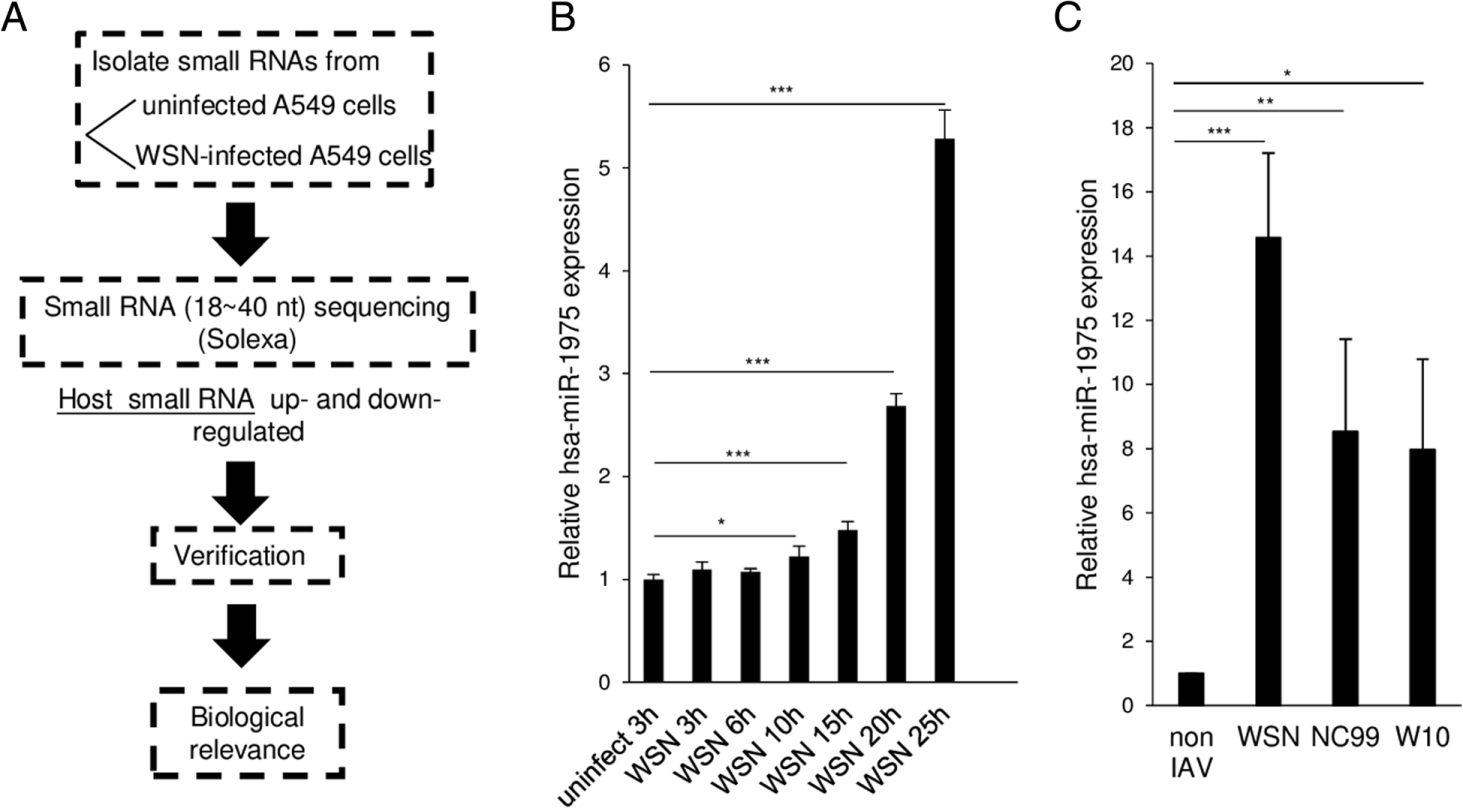
Expression of hsa-miR-1975 in A549 cells after influenza virus infection. **a** Outline of the identification of small RNAs involved in influenza A virus infection. A549 cells were infected with influenza virus (A/WSN/33) at an MOI of 5 or uninfected. At 25 h p.i.(hours post infection), small RNAs ranging from 18 to 40 nucleotides were collected and processed for RNA sequencing. The interesting small RNA was identified and selected for further validation. **b** A549 cells were infected with WSN (MOI = 5) and harvested at different time points for RT-qPCR analysis. The RNA levels of hsa-miR-1975 were normalized by U24 RNA. Values represent the mean ± SD of three independent experiments. **c** A549 cells were infected with different strains of influenza A virus (MOI = 0.01) for 40 h and then harvested for RT-qPCR analysis. The RNA levels of hsa-miR-1975 were normalized by U24 RNA. Values represent the mean ± SD of four independent experiments. Statistical comparisons between groups by one-way ANOVA with Bonferroni correction for multiple comparisons (**b** and **c**). Statistically significant differences are indicated as follows: *P < 0.05, **P < 0.01, and ***P < 0.001

To search for the small RNAs likely participating in IAV infection, we performed deep sequencing of small RNAs from influenza virus (A/WSN/33)-infected and uninfected A549 cells, a human lung epithelial cell line (Fig. 1a). We used bioinformatics analysis to identify several host small RNAs either up-regulated or down-regulated at 25 h post-infection (p.i.). At least 5 RNA species were up-regulated by more than two folds in the infected cells, while 5 other RNA species were down-regulated to similar extent (Additional file 1: Figure S1). Notably, among these ten small RNAs, miR-886, miR-378 and miR-151 had been previously reported to be associated with influenza virus infection, strengthening the reliability of our method [8, 27, 28]. Most of these RNA species are microRNAs. However, the most highly up-regulated small RNA in our screen was a Y5-derived small RNA, hsa-miR-1975, which does not belong to microRNA. In subsequent studies we focused on this RNA.

To investigate the expression profile of hsa-miR-1975 upon IAV infection, we measured the expression levels of hsa-miR-1975 by reverse transcription-quantitative polymerase chain reaction (qPCR) at several time points. The relative amounts of hsa-miR-1975 exhibited substantial increase beginning at 10 h p.i. (hours post infection) (Fig. 1b). The abundance of hsa-miR-1975 increased up to 5 folds at 25 h p.i. (Fig. 1b). To elucidate whether the increase of hsa-miR-1975 is specific to WSN strain, A549 cells were infected with different strains of influenza viruses, including A/WSN/33 (H1N1), influenza A/New Caledonia/20/1999 (NC99) (H1N1) and A/Wisconsin/67/2005 (W10) (H3N2), at a multiplicity of infection (MOI) of 0.01 for 40 h. The expression of hsa-miR-1975 increased significantly after A549 cells were infected with each of the three virus strains (Fig. 1c), suggesting that the increase of hsa-miR-1975 is a general phenomenon associated with the infection by most of the influenza virus strains.

### Y5RNA is processed to hsa-miR-1975 during cell apoptosis

Human Y RNAs are rapidly and specifically cleaved into small RNAs of 24 to 34 nucleotides in a caspase-dependent manner, and this occurs under the duress of a range of apoptotic stimuli [29, 30]. Since influenza virus infection induces apoptosis [16], we examined whether the enhanced expression of hsa-miR-1975 is related to apoptosis upon influenza virus infection. For this purpose, a pan-caspase inhibitor, carbobenzoxy-valyl -alanyl-aspartyl-[O-methyl]-fluoromethylketone (Z-VAD-FMK), was used to inhibit both intrinsic and extrinsic apoptosis pathways. We provided evidence showing cell apoptosis occurred at 24 h post infection at MOI = 0.1 (Additional file 2: Figure S2). Adding VAD partially mitigated levels of apoptosis.

**Fig. 2:**
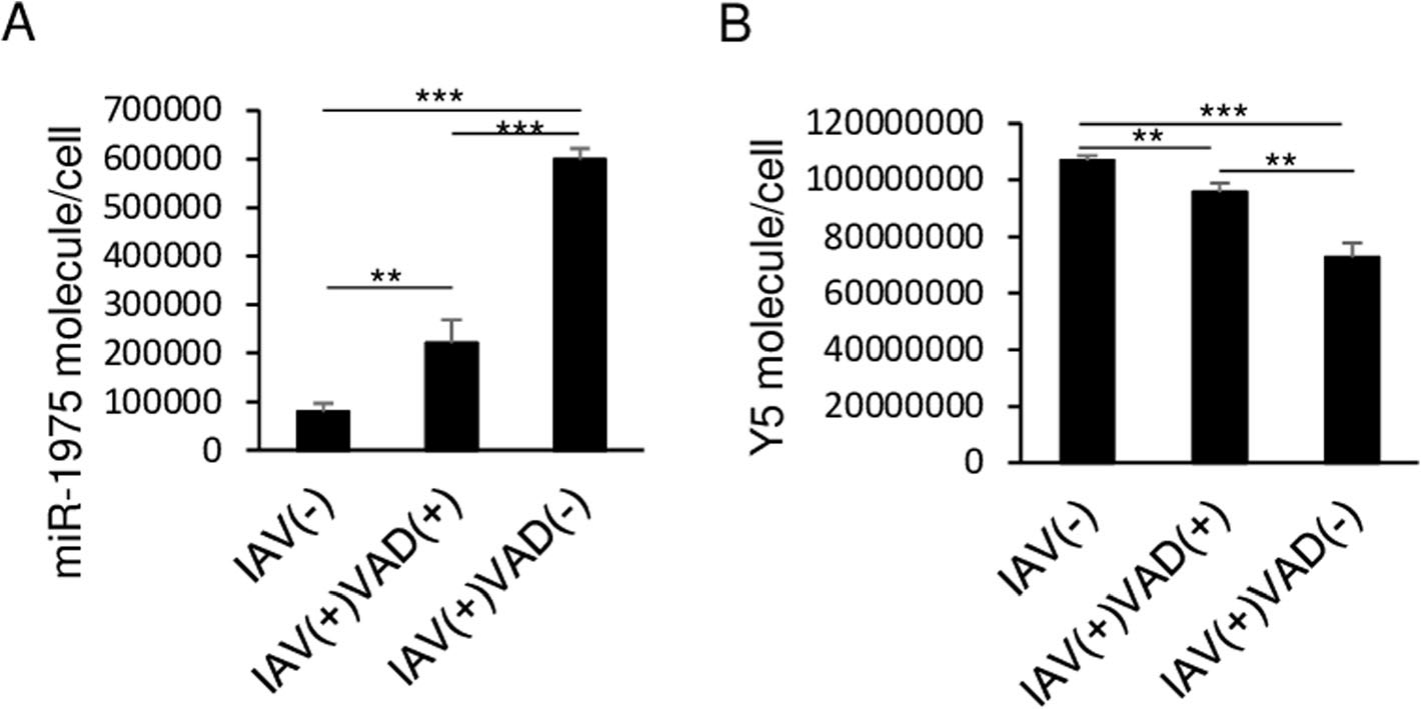
Biogenesis of hsa-miR-1975 during apoptosis. A549 cells were treated with either mock or VAD (a pan-caspase inhibitor) for 6 h and kept the treatment during virus infection and then infected with IAV (WSN) (MOI = 0.1) for 24 h or uninfected. **a** Numbers of hsa-miR-1975 molecules per cell in different groups were compared. **b** Numbers of human Y5 molecules in different groups were compared. Values represent the mean ± SD of three independent experiments. Statistical comparisons between groups by one-way ANOVA with Bonferroni correction for multiple comparisons. *P < 0.05, ***P < 0.001

In order to address the real differences in Y5 and miR-1975, we quantified the amounts of Y5 and miR-1975 by constructing the standard equations of serial dilution of miR-1975 and Y5 mimic (Additional file 3: Figure S3). The amounts of miR-1975 and Y5 are shown in Fig. 2. Influenza WSN virus infection induced an 8-fold increased expression of hsa-miR-1975; this increase was partially blocked by the presence of VAD, suggesting that the decline of hsa-miR-1975 expression during the viral infection was a result of inhibition of apoptosis (Fig. 2a). Correspondingly, the amount of human Y5 RNA decreased in the infected A549 cells, and this decrease was partially blocked by the treatment of VAD (Fig. 2b). IAV replication is suppressed after adding VAD (Additional file 4: Figure S4). This finding is in line with previous study indicating that blockage of apoptosis reduce influenza virus production [16]. About 5.2 × 10^5^ hsa-miR-1975 molecules are produced while 3.4 × 10^7^ Y5 molecules are consumed in a single A549 cell after WSN infection at a MOI = 0.1 for 24 h. Since hsa-miR-1975 has been demonstrated to be derived from Y5RNA [31], we infer that a small part of consumed human Y5 RNA is processed into hsa-miR-1975 after infection. This phenomenon is mitigated after adding an apoptosis inhibitor.

### hsa-miR-1975 mimic inhibits influenza virus replication

The increased expression of hsa-miR-1975 during IAV infection suggests that it may play some roles in IAV life cycle. To address this possible role, we transfected a synthetic hsa-miR-1975 mimic into cells before IAV infection and collected cell lysates at different time points after influenza virus infection. We showed the levels of endogenous and exogenous amount of miR-1975 (Additional file 5: Figure S5). The levels of miR-1975 increased in cells after transfected with miR-1975 mimic is about 300 times of the levels of miR-1975 increased that sorely elevated by virus infection.

**Fig. 3:**
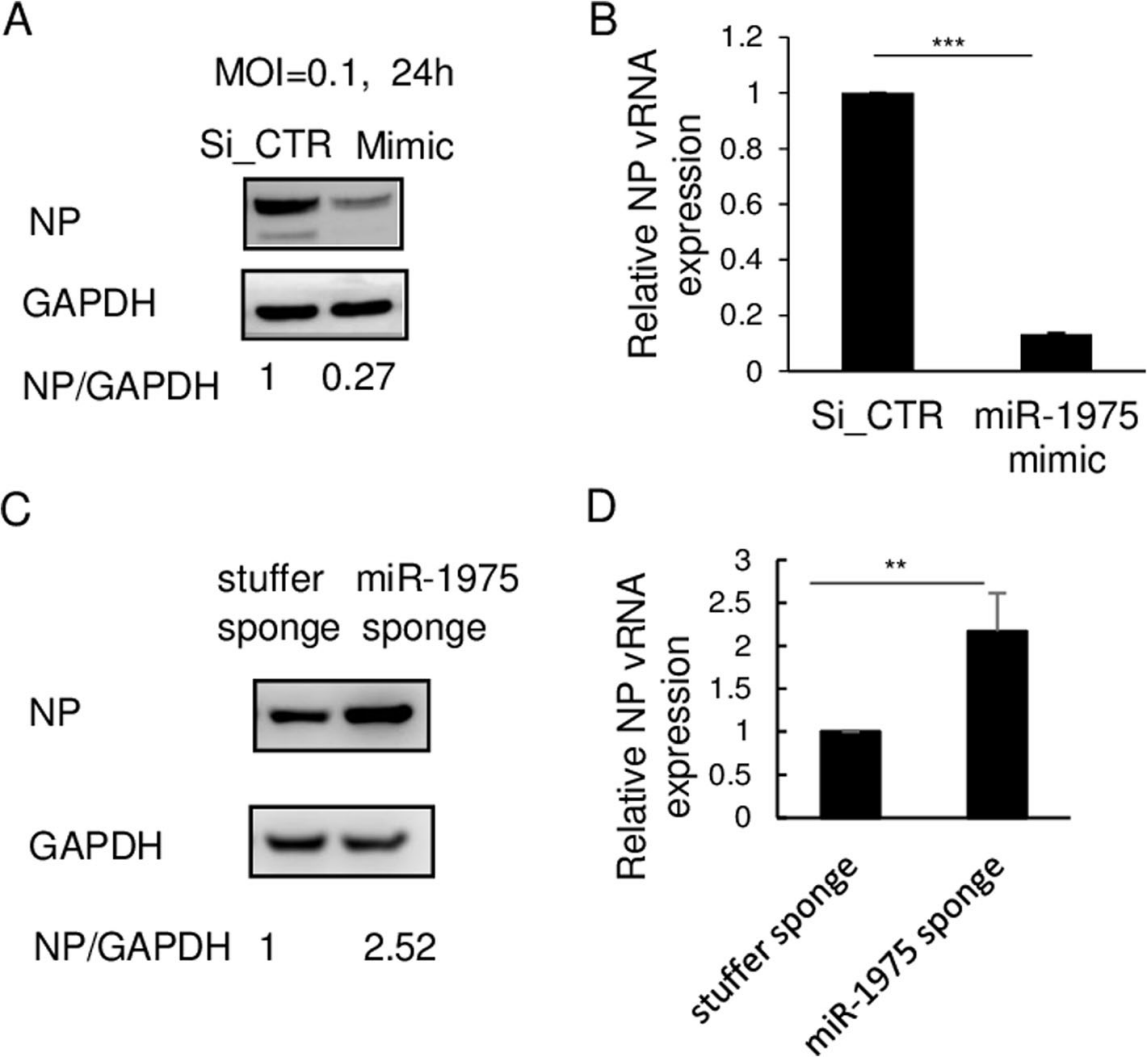
The effects of hsa-miR-1975 mimic and sponge on WSN replication. **a** A549 cells were transfected with control SiRNA (Si_CTR) or hsa-miR-1975 mimic (mimic) and then infected with WSN (MOI = 0.1). At 24 h after infection, cells were harvested for immunoblotting of viral NP and cellular GAPDH proteins. The band intensities were quantified, and the relative NP/GAPDH ratios are shown below the blots. **b** A549 cells were transfected with control SiRNA (Si_CTR) or hsa-miR-1975 mimic (miR-1975 mimic) and then infected with WSN (MOI = 0.1). At 24 h p.i., cells were harvested and processed for RT-qPCR analysis. The NP expression levels were normalized by GAPDH. **c** and **d**, A549 cells were either transfected with control stuffer sponge or miR-1975 sponge and then infected with WSN (MOI = 0.1) for 24 h. Western Blots of viral NP and cellular GAPDH were performed. The numbers below Western Blot are NP protein ratio normalized by GAPDH (**c**). Relative NP vRNA levels were assessed by RT-qPCR and normalized by GAPDH (**d**). Values represent the mean ± SD of three independent experiments (**b** and **d**). Statistical comparisons between groups by Student’s *t* test (**b** and **d**). **P < 0.01, ***P < 0.001

We found that the amount of influenza viral nucleoprotein (NP) was significantly lower in the cells transfected with hsa-miR-1975 mimic, as compared with control SiRNA or scramble (Fig. 3a and Additional file 6: Figure S6a). It was further confirmed by measuring the amount of viral RNA (vRNA) segment 5 encoding NP by RT-qPCR (Fig. 3b and Additional file 6: Figure S6b). To ascertain the importance of miR-1975 in inhibiting IAV replication, we also measured the yield of virions after manipulations with hsa-miR-1975 mimic by plaque assay. The progeny virus titer was significantly reduced after hsa-miR-1975 transfection compared to that after control SiRNA or scramble transfection (Additional file 6: Figure S6c). To further verify the role of hsa-miR-1975, a hsa-miR-1975 sponge, which blocks the function of hsa-miR-1975 by complementary binding to most of hsa-miR-1975 sequence, was transfected into cells. Western blot and RT-qPCR results showed a significant increase of viral protein and RNA amounts in hsa-miR-1975 sponge-transfected cells, as compared to stuffer sponge control (Fig. 3c and d). Collectively, these results indicated that increased function of hsa-miR-1975 (by “mimic”) caused the suppression of viral protein and RNA levels, whereas the decreased function (by “sponge”) enhanced their production, suggesting that hsa-miR-1975 plays a negative role for IAV replication.

### hsa-miR-1975 exerts antiviral effect through stimulating interferon production

**Fig. 4:**
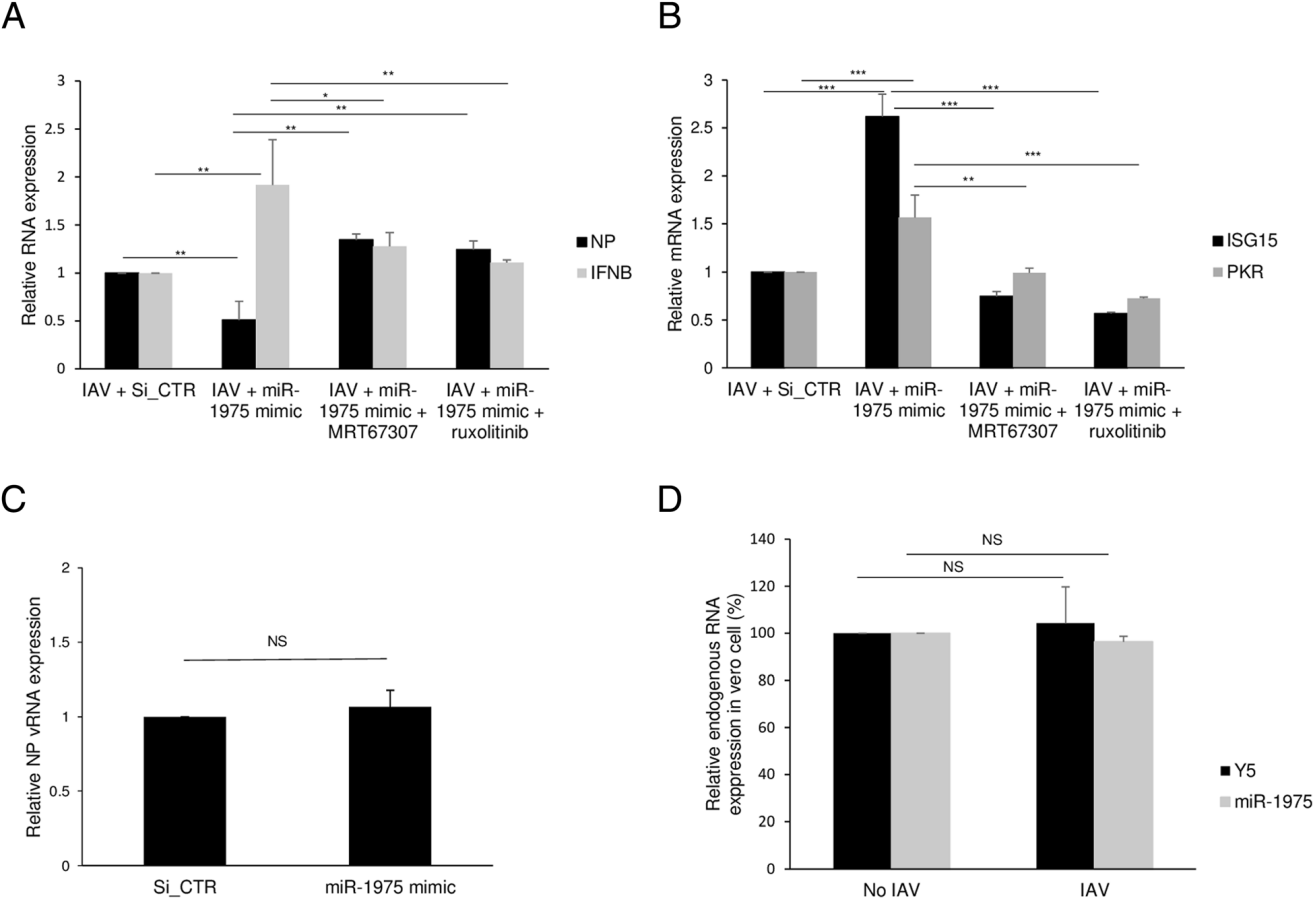
Expression of *IFNB* after hsa-miR-1975 mimic transfection. **a** A549 cells were not treated, pretreated with 1 μM MRT67307 for 30 min or pretreated with 4 μM ruxolitinib for 24 h. The cells were then transfected with control SiRNA (Si_CTR) or hsa-miR-1975 mimic as indicated. At 48 h post-transfection, A549 cells were infected with WSN (MOI = 1) for 6 h and then harvested. Cellular RNAs were extracted and measured by RT-qPCR. The levels of NP vRNA and IFNB mRNA were normalized by GAPDH mRNA. Values represent the mean ± SD of three independent experiments. **b** Expression of ISG15 and PKR mRNA were normalized by GAPDH mRNA from A549 cells that were treated as mentioned in (**a**). Values represent the mean ± SD of three independent experiments. **c** Vero cells were transfected with control SiRNA (Si_CTR) or hsa-miR-1975 mimic. At 48 h post-transfection, Vero cells were infected with WSN at a MOI of 1 for 6 h and then harvested. Cellular RNAs were extracted and measured by RT-qPCR. The levels of NP vRNA were normalized by GAPDH mRNA. Values represent the mean ± SD of three independent experiments. **d** Vero cells were infected with WSN at a MOI of 1 for 6 h and then harvested. Cellular RNAs were extracted and measured by RT-qPCR. Levels of endogenous miR-1975 and Y5 expression were normlized by U24. Values represent the mean ± SD of three independent experiments.Statistical comparisons between groups by one-way ANOVA with Bonferroni correction for multiple comparisons (**a** and **b**) and Student’s *t* test (**c** and **d**). *P < 0.05, **P < 0.01, and ***P < 0.001

The above result that hsa-miR-1975 is not required for IAV replication but instead inhibits viral replication implies that it may be involved in cellular antiviral responses. Given that small RNAs have potential to induce interferon response, we postulated that hsa-miR-1975 may inhibit virus replication by stimulating interferon production [17, 32, 33]. We therefore examined whether introduction of hsa-miR-1975 mimic alters the expression of *IFNB* mRNA. We used MRT67307 and ruxolitinib to testify the hypothesis. MRT67307 is a dual inhibitor of IKKε and TBK1 [34], both of which regulate IFNB expression. Ruxolitinib is a JAK1 and JAK2 inhibitor. Pretreating cell with either MRT67307 or ruxolitinib resulted in downregulation of IFNB, ISG15 and PKR. This contributed to productive IAV replication (Additional file 7: Figure S7a and b). Next, we examined whether hsa-miR-1975-induced interferon gene expression exhibits antiviral effect. After transfection with hsa-miR-1975 mimic or control SiRNA, the cells were infected with WSN virus and then harvested at 6 h p.i. We found that the hsa-miR-1975 mimic induced the expression of *IFNB* gene more significantly than the control siRNA; correspondingly, the viral NP amount was reduced by hsa-miR-1975 (Fig. 4a). We found the effect of miR-1975-dependent suppression of IAV is lost after adding MRT67307 or ruxolitinib (Fig. 4a). miR-1975-dependent interferon stimulating gene activation, such as ISG15 and PKR, was attenuated after adding MRT67307 or ruxolitinib (Fig. 4b). These results imply that activation of IFNB gene expression may account for the effect of hsa-miR-1975. To further assess whether hsa-miR-1975 inhibits IAV replication mainly through the interferon pathway, we transfected hsa-miR-1975 mimic into Vero cells, which are deficient in interferon production [35]. *IFNB* mRNA was not detected in Vero cells before and after IAV infection (data not shown). Significantly, hsa-miR-1975 mimic did not suppress IAV in Vero cells (Fig. 4c). We provided evidence showing that similar changes at endogenous miR-1975 and Y5 RNA levels in Vero cells upon IAV infection (Fig. 4d). Together, these data demonstrated that hsa-miR-1975 induces interferon production, suggesting its role in host cell’s antiviral response.

### hsa-miR-1975 is packaged into exosomes after IAV infection

**Fig. 5:**
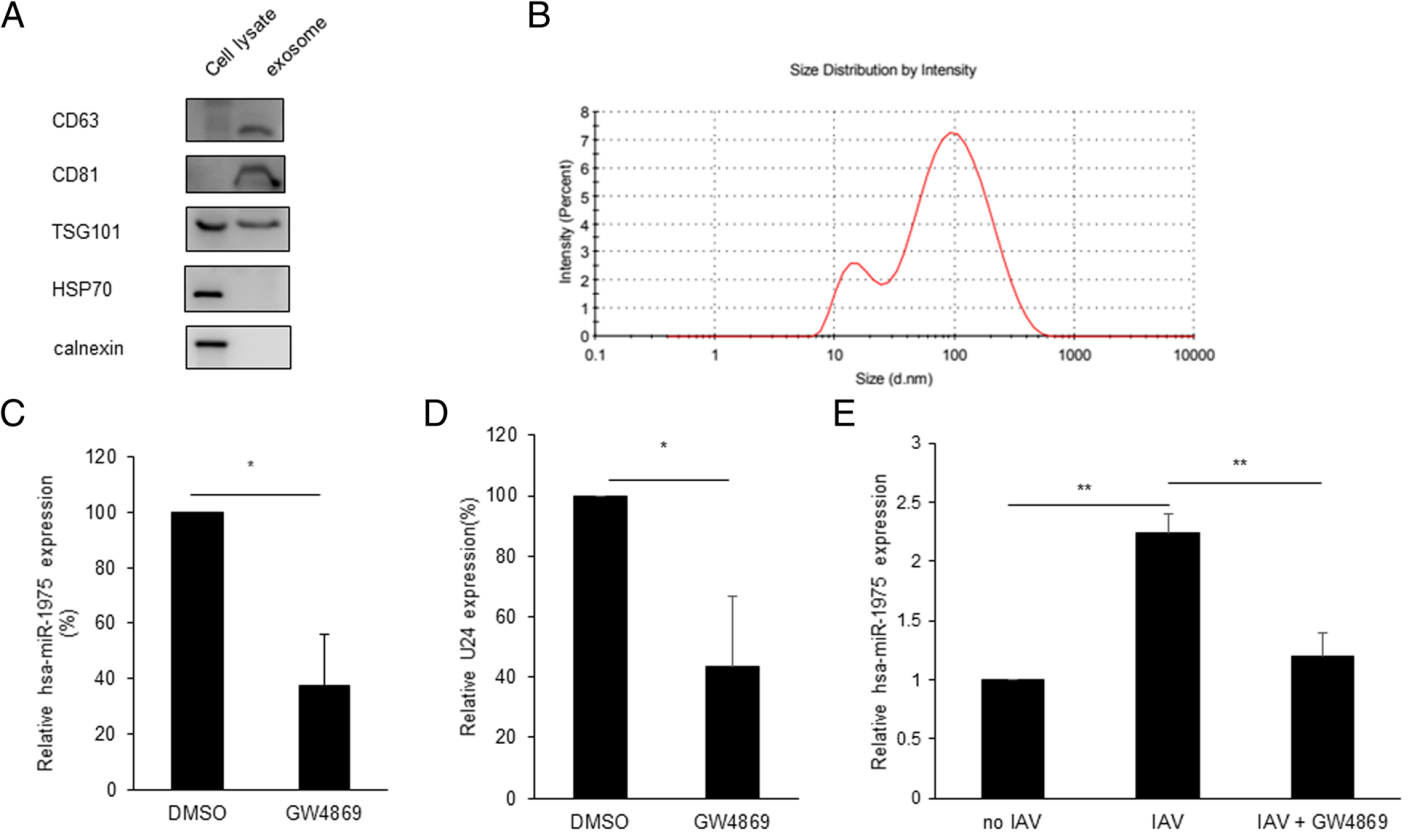
hsa-miR-1975 is packaged into exosomes. **a** Characterization of exosomes by Western Blot analysis. The A549 cell lysates and exosomes were immunoblotted with the indicated antibodies. **b** Exosomes pellets were diluted in 500 μL PBS. Size of exosomes were determined by using the dynamic light scattering technique (Zetasizer Nano ZS, Malvern Instruments, UK). **c** Relative expression of hsa-miR-1975 in exosomes after treating with 10 μM GW4869 or DMSO for 24 h. Values represent the mean ± SD of three independent experiments. **d** Relative expression of U24 in exosomes after treating with 10 μM GW4869 or DMSO for 24 h. Values represent the mean ± SD of three independent experiments. **e** Assessment of relative expression of hsa-miR-1975 in exosomes without WSN infection,with WSN infection (MOI = 1) for 24 h and with WSN infection (MOI = 1) and 10 μM GW4869 treatment for 24 h by RT-qPCR. Values represent the mean ± SD of three independent experiments. Statistical comparisons between groups by Student’s *t* test (**c**-**d**) and one-way ANOVA with Bonferroni correction for multiple comparisons (**e**). *P < 0.05, **P < 0.01 and ***P < 0.001

It has been recognized that exosomal transfer of small RNA is an important route for intercellular communication [14]. Small RNAs containing certain specific sequences, called exosomal motifs, are prone to be packaged into exosomes; on the contrary, certain cellular-bound motifs exist in small RNAs prone to be retained in cells [15]. We found that the nucleotide sequence of hsa-miR-1975 contains exosomal motifs CCCA and UGAC, indicating that hsa-miR-1975 may exert its biologic function through excreting in exosomes [15]. To confirm this possibility, we first determine whether hsa-miR-1975 could excrete to extracellular space by examining the intracellular and extracellular distribution of hsa-miR-1975. About half amounts of hsa-miR-1975 were excreted in the extracellular space while less than 0.5% of Y5 and U24 small RNAs were excreted in the extracellular space regardless of viral infection (Additional file 8: Figure S8a and b). This finding prompted us to speculate that hsa-miR-1975 possibly exerts its function through secreting to extracellular space. The exosome fractions were then isolated from cells and characterized by Western blotting with specific markers (Fig. 5a). As expected, the exosomal markers, including CD63, CD81 and TSG101 [36], were enriched in exosomes. Calnexin, one of the ER markers, and the cellular ubiquitously expressed heat shock protein 70 (HSP70) were undetected in exosomes. Sizes of exosomes are heterogenous and ranges from 30 to 120 nm [37]. We further showed that the isolated exosomes had an average particle size of 53 nm, as determined by dynamic light scattering (Fig. 5b). To validate that hsa-miR-1975 is packaged into exosomes, A549 cells were treated with GW4869, a drug known to hinder exosome biogenesis by blocking neutral sphingomyelinase 2 [38, 39]. Poly(I:C), which can induce cell apoptosis, was also added to stimulate the production of hsa-miR-1975 in cells [11]. The results showed that hsa-miR-1975 in the exosomes was significantly decreased from the culture medium of GW4869-treated cells as compared to that of DMSO-treated cells, further indicating that hsa-miR-1975 is contained within exosomes (Fig. 5c). We found that GW4869 promotes IAV replication (Additional file 9: Figure S9). This is consistent with the idea that the inhibition of transferring antiviral molecule enclosed in exosomes facilitates viral replication.

We next examined whether IAV infection affects the levels of hsa-miR-1975 in exosomes. Exosomes pellets were treated with RNase before RNA isolation to make sure that hsa-miR-1975 is contained within exosomes rather than contaminants on the outside of exosomes. We chose another small RNA, U24, as a control for analysis of exosomal hsa-miR-1975 because U24 has been shown to be packaged into exosomes [40]. Furthermore, we showed that U24 in the exosomes was reduced to 40% from the culture medium of GW4869-treated cells as compared to that of DMSO-treated cells (Fig. 5d). The levels of U24 reduction were similar to miR-1975 reduction in exosomes from GW4869-treated cells (Fig. 5c and d). This indicates that the exosome package efficiencies of U24 and miR-1975 are similar. We showed that the relative levels of hsa-miR-1975 were increased in exosomes from WSN-infected A549 cells compared to that from the uninfected cells (Fig. 5e). The abundance of hsa-miR-1975 dropped after adding GW4869 (Fig. 5e). Taken together, these results suggest that hsa-miR-1975 can be packaged into exosomes.

### Exosomal hsa-miR-1975 exerts antiviral effect in recipient cells

**Fig. 6:**
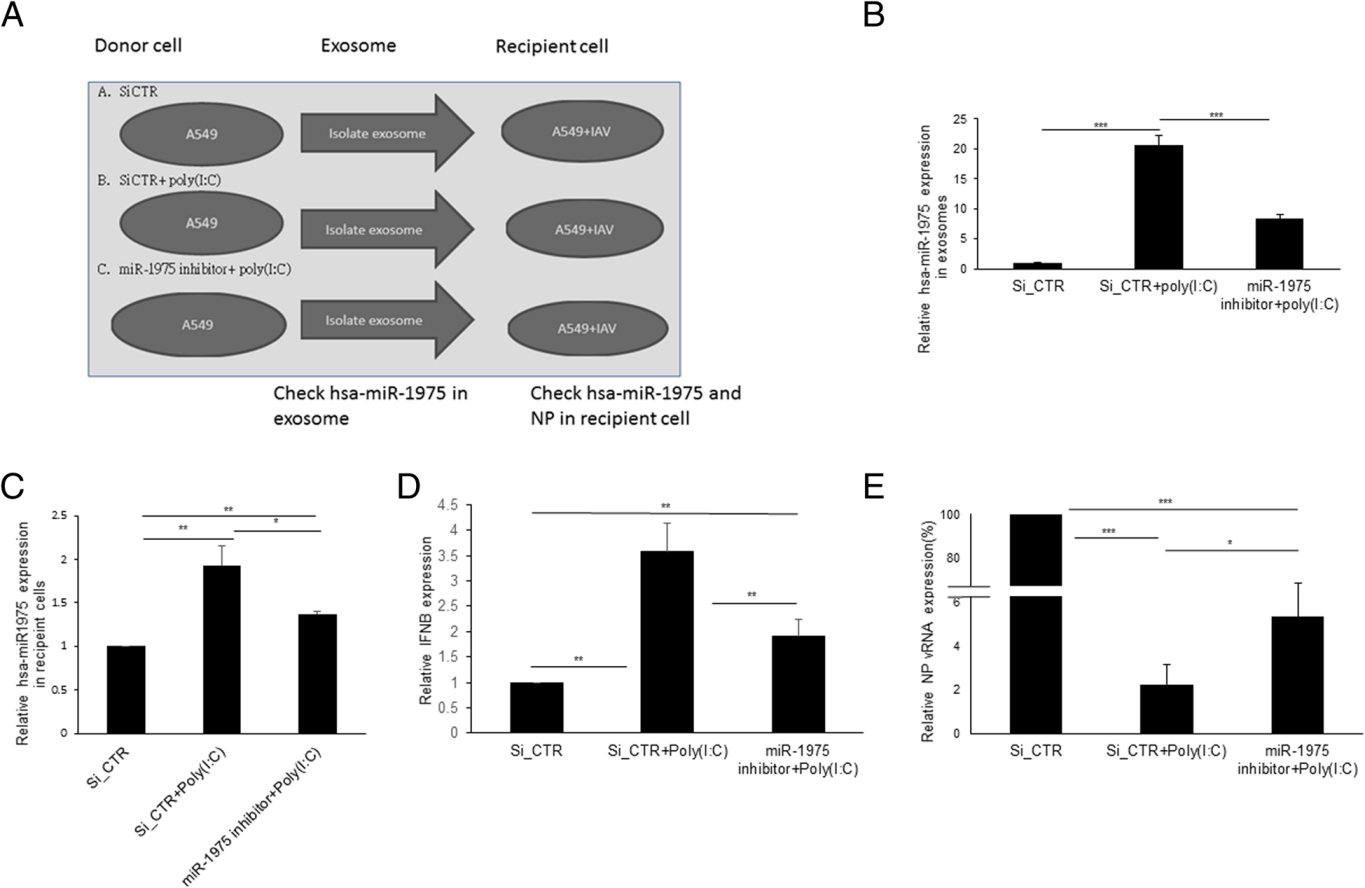
Exosome-delivered hsa-miR-1975 suppresses influenza virus replication. **a** Illustration of the experimental design to study the functional role of exosomally transferred hsa-miR-1975. Donor cells were transfected with control siRNA (Si_CTR) or hsa-miR-1975 inhibitor and then treated with mock or Poly(I:C). **b** Expression of hsa-miR-1975 in exosomes from donor cells after treateing with RNases was assessed by RT-qPCR. Values represent the mean ± SD of three independent experiments. **c** Expression of hsa-miR-1975 in recipient cells after receiving exosomes from donor cells as illustrated in (**a**) and then infected with WSN (MOI = 1) for 6 h. U24 was used for normalization of expression. Values represent the mean ± SD of three independent experiments. **d** Expression of IFNB mRNA in recipient cells after receiving exosomes from donor cells that were treated as mentioned in (**c**). GAPDH was used for normalization of expression. **e** The cellular RNAs were harvested and assessed by RT-qPCR for quantification of NP vRNA. GAPDH was used for normalization of expression. Expression of NP vRNA in recipient cells after receiving exosomes from donor cells that were treated as mentioned in (**c**). Values represent the mean ± SD of three independent experiments. Statistical comparisons between groups by one-way ANOVA with Bonferroni correction for multiple comparisons (**b**-**e**). *P < 0.05, **P < 0.01, and ***P < 0.001

After examining the existence of hsa-miR-1975 in exosomes, we investigated whether exosomal hsa-miR-1975 would be transferred among cells. The design of experiments was illustrated in Fig. 6a. To induce the expression of hsa-miR-1975, Poly(I:C), known to enhance YsRNAs production, was used [11]. Exosomes were isolated from three groups of donor cells and then added to recipient cells and incubated for 24 h. The recipient cells were further infected with WSN for 6 h and the amount of the NP vRNA was then quantified by RT-qPCR. The concentration of exosomal protein content had no significant difference among the three groups (Additional file 10: Figure S10). The expression of exosomal miR-1975 was up-regulated when A549 cells were treated with Poly(I:C) and this effect was significantly attenuated when donor cells were pretreated with hsa-miR-1975 inhibitor (Fig. 6b). Subsequently, we treated recipient A549 cells with exosomes from these three groups of donor cells and then infected the recipient cells with WSN virus. The results showed that Poly(I:C) increased the amount of hsa-miR-1975 in recipient cells, but this increase was attenuated by the hsa-miR-1975 inhibitor (Fig. 6c). Exosomes from poly(I:C)-treated cells are rich in interferon-stimulating molecules (Fig. 6d). We proposed that miR-1975 is one of them. To elucidate the antiviral significance of exosomal miR-1975, we inhibited the transfer of miR-1975 in recipient cell by adding complementary nucleotides to donor cells. We observed a decreased antiviral and interferon-stimulating effect of poly(I:C)-induced exosomes after inhibiting the transfer of miR-1975 to exosomes (Fig. 6d and e). Together, all of the aforementioned findings suggest that hsa-miR-1975 can exert antiviral effect through excreting into exosomes.

## Discussion

98.5% of the human genome contains non-coding sequences [41], and small non-coding RNAs (sncRNAs) make up a significant portion of these transcriptomes. Many of these sncRNAs regulate gene expression and possibly mediate intercellular physiological signals [14, 36, 42, 43]. Human Y RNAs range in size from 83 to 112 nucleotides and fold into stem-loop secondary structures [44, 45]. hsa-miR-1975, located at position 51–75 of Y5 RNA, consisting of 3′ end of stem domain and internal loop domain of human Y5 RNA. In contrast to traditional miRNA biogenesis, YsRNAs neither associate with Argonaute proteins nor are dependent on Dicer [11, 46, 47], implying the difficulty of identifying the potential mRNA targets of hsa-miR-1975 by using immunoprecipitation with AGO2 antibody.

In this study, we explored the biogenesis of hsa-miR-1975. We found that the expression levels of hsa-miR-1975 were increased at late stages of influenza viral life cycle (Fig. 1b), in accordance with the onset of late apoptotic events. Chakrabortty et al. demonstrated that a 23 or 31 nucleotide processed fragments of Y5 from cancer cells induced cell death in primary cells through triggering differential expression of genes associated with the *FAS*/*TGF*-β-*Smad2/3* apoptotic pathway [13]. Nineteen of 25 nucleotides of hsa-miR-1975 sequence are complementary to the 23- or 31-nucleotide processed fragments of Y5 described by Chakrabortty et al.

The fold increased for miR-1975 at 25 h p.i. was about 20 fold based on the RNA sequence. It increased for 5 fold when we used stem-loop RT-PCR to verify the significance. The different methods used by the two experiments account for the differences in fold change. We further validated the increase of miR-1975 after influenza virus replication is not specific to definite species. In Fig. 1c we infected cell for 40 h with 0.01 MOI of virus because the titer of NC99 and W10 influenza viruses are lower and it will not cause significant increase of miR-1975 within 24 h. When we infected WSN with high MOI for more than 25 h, there is significant cell death and few cells could be collected for analysis. Therefore, we infected A549 cells with lower MOI and longer time.

To clarify the relationship between hsa-miR-1975 and cell viability, we transfected hsa-miR-1975 mimic or inhibitor into cells and examined the cell viability of transfected cells. We found that neither hsa-miR-1975 mimic nor its inhibitor influenced cell viability regardless of influenza virus infection (Additional file 11: Figure S11). Rutjes et al. demonstrated that Y RNA is cleaved and subsequently truncated to YsRNA (29). This process could be inhibited by anti-apoptosis protein Bcl-2 and the caspase inhibitor, implying that nucleolytic activity of Y RNA is activated downstream of caspase cascade [29]. We corroborated that the biogenesis of hsa-miR-1975 is a consequential event upon influenza virus-induced apoptosis by demonstrating that hsa-miR-1975 was decreased in the presence of a pan-caspase inhibitor (Fig. 2a). Nevertheless, the causal relationship between Y RNA and hsa-miR-1975 needs additional confirmation.

Thus far, the role of Y RNA in viral infection has not been addressed. Initially, we thought that since hsa-miR-1975 expression increased during viral replication, it is most likely a positive factor involved in viral replication. However, to our surprise, its presence inhibited viral replication instead. We demonstrated that the viral inhibition effect could not only be found at 24 h but also be found at early stage of infection. We used MOI 1 and infected A 549 cells for 6 h because the difference is not significant with MOI 0.1 at 6 h. Thus, miR-1975 is most likely a cellular factor in the host’s antiviral defense. To further investigate this issue, we overexpressed hsa-miR-1975 mimic in A549 cells. We observed an upregulation of *IFNB* and diminished abundance of NP in hsa-miR-1975-transfected cells as compared to control SiRNA or mock-transfected cells (Fig. 4b). Lack of antiviral effect of hsa-miR-1975 mimic in interferon-deficient Vero cells indicated that interferon plays a pivotal role in the context of antiviral immunity related to hsa-miR-1975 (Fig. 4d). Goldgraben et al. postulated that differing lengths, rather than sequence, of RNA was responsible for differential interferon responses [48]. We have addressed this issue by comparing the antiviral effect and interferons-stimulating effect of hsa-miR-1975 mimic and a scrambled miR-1975 RNA which is also a 25-mer RNA with the same GC ratio. The results indicated that the sequence of hsa-miR-1975 play vital role in stimulating interferon and inhibiting IAV replication (Additional file 6: Figure S6a, 6b and Additional file 12: Figure S12).

YsRNA molecules have been detected in sera of healthy people [31]. It is intriguing to notice that YsRNA existed in sera wherein the concentrations of RNases are estimated at several hundred nanograms/ml [49, 50]. There are several explanations to this sequence stability. First, Ro60 and La proteins are bound to YsRNA, thereby preventing YsRNA from RNase digestion [29, 31]. Another mechanism is the natural encapsulation of these molecules in vesicles [51]. Y5-derived small RNA had been demonstrated to be packaged into exosomes [13]. We demonstrated that hsa-miR-1975 exerted its function also through packaging into exosomes. These results were expected on the basis of hsa-miR-1975 containing specific sequences which are prone to be secreted in exosomes [15].

By examining the expression of hsa-miR-1975 and influenza virus NP in recipient cell after receiving exosomes derived from control SiRNA or hsa-miR-1975 inhibitor treated donor cells, we underscored its biologic relevance in cell-to-cell communication. Viral replication in recipient cell was inhibited drastically after receiving exosomes derived from Poly(I:C)-treated donor cells (Fig. 6d). We postulated that exosomes derived from Poly(I:C)-treated donor cells contain proteins or small RNAs that would drive signal transduction to exert antiviral effect. Importantly, by inhibiting hsa-miR-1975 in donor cells, we demonstrated a downregulation of hsa-miR-1975 and enhancement of influenza virus replication in recipient cells (Fig. 6c and d). Together, these results suggest a functional role of exosomal hsa-miR-1975 in defense responses against viral infections.

## Conclusions

**Fig. 7:**
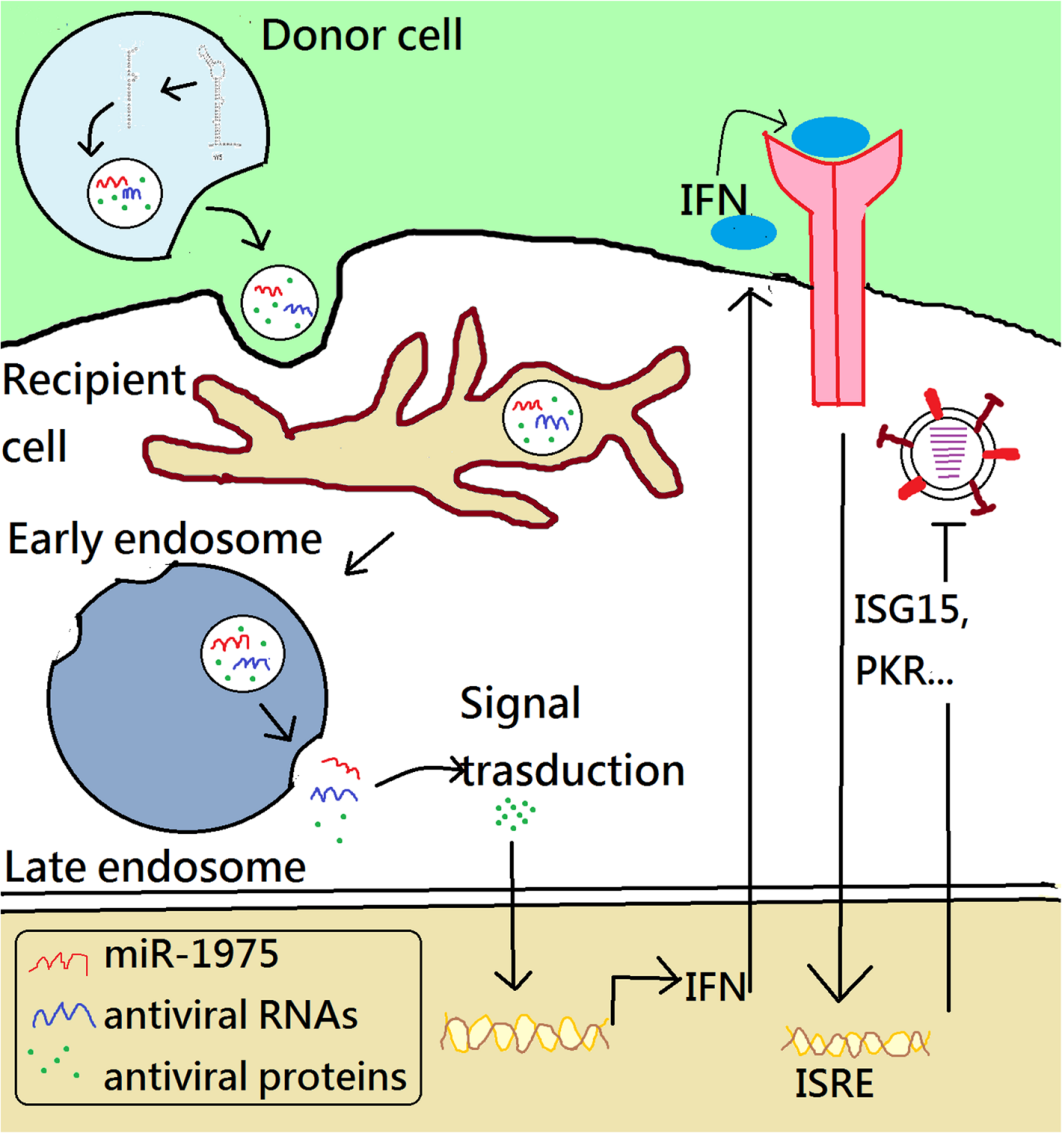
The mechanism of how miR-1975 inhibits viral replication. After virus infection, cells undergo apoptosis. The Y5 RNA is degraded to a Y5 RNA-derived small RNA, hsa-miR-1975, during apoptosis. Hsa-miR-1975 and other molecules are packaged into exosomes and then uptaken by neighboring cells. Exosomally delivered hsa-miR-1975 together with other antiviral molecules fuels the production of interferon in recipient cells. Interferons transfer signal to interferon stimulating gene, containing interferon-sensitive response element (ISRE), to inhibit viral replication

Here, we propose a mechanism by which hsa-miR-1975 inhibits IAV replication (Fig. 7). Infection with influenza virus causes cell apoptosis, leading to biogenesis of hsa-miR-1975. hsa-miR-1975 is subsequently delivered into exosomes and engulfed by neighbor cells. Hsa-miR-1975, together with other antiviral proteins or nucleotides, induces interferon production and thereby inhibits influenza virus replication when viruses invade these recipient cells. Our study not only revealed the role of hsa-miR-1975 in host innate immunity against influenza virus but also contributed to the understanding of the biology of Y5 RNA-derived small RNAs.

## Additional files

Additional file 1: **Figure S1.** Major up-regulated and down-regulated small RNAs in IAV-infected cells. (a) Top 5 up-regulated small RNAs. (b) Top 5 down-regulated small RNAs. Cellular small RNAs expression patterns in A549 cells infected with WSN were compared with those of uninfected A549 cells. Differentially expressed small RNAs with a fold change > 2 are described. (TIF 55 kb)

Additional file 2: **Figure S2.** Western Blot of Caspase3 and GAPDH after influenza infection and treated with VAD. A549 cells were treated with either mock or 20 μM VAD (a pan-caspase inhibitor) for 6 h and kept the treatment during virus infection and then infected with IAV (WSN) (MOI = 0.1) for 24 h or uninfected. WB of caspase3 and GAPDH was performed. The numbers below Western Blot are cleaved caspase3 protein ratio normalized by GAPDH. (TIF 177 kb)

Additional file 3: **Figure S3.** (a) Standard equation of log_e_ (number of miR-1975 molecules) and Ct value of miR-1975. (b) Standard equation of log_e_ (number of Y5 molecules) and Ct value of Y5. (TIF 129 kb)

Additional file 4: **Figure S4.** Inhibition of apoptosis by VAD promotes IAV replication. A 549 cells were treated with either mock or VAD (a pan-caspase inhibitor) for 6 h. Relative expressions of NP viral RNA in A549 cells were compared. Values represent the mean ± SD of three independent experiments. Statistical comparisons between groups by Student’s *t* test. *P < 0.05. (TIF 7 kb)

Additional file 5: **Figure S5.** Levels of endogenous and exogeneous miR-1975 in A549 cells after infected with WSN and miR-1975 mimic transfection. A549 cells were transfected with control SiRNA (Si_CTR) or miR-1975 mimic and then infected with WSN (MOI = 0.1) or not infected. At 24 h p.i., cells were harvested and processed for stem-loop RT-qPCR analysis of miR-1975. Values represent the mean ± SD of three independent experiments. (TIF 21 kb)

Additional file 6: **Figure S6.** Transfecting hsa-miR-1975 mimic reduces WSN replication. (a) and (b), A549 cells were transfected with control SiRNA (Si_CTR), scramble miR-1975 (scramble) and hsa-miR-1975 mimic and then infected with WSN at MOI of 1. At 6 h p.i., cells were harvested for immunoblotting of viral NP and cellular GAPDH proteins (a) or RT-qPCR of NP vRNA expression (b). The band intensities were quantified, and the relative NP/GAPDH ratios are shown below the blots (a). The relative NP expressions upon WSN infection were assessed by RT-qPCR. The expression levels were normalized by GAPDH (b). (c) A549 cells were infected with influenza WSN at an MOI of 0.1. The supernatants were collected at 24 h p.i and used for determining the viral titer by plaque assay in MDCK cells. Values represent the mean ± SD of three independent experiments. Statistical comparisons between groups by one-way ANOVA with Bonferroni correction for multiple comparisons. *P < 0.05 and **P < 0.01. (TIF 97 kb)

Additional file 7: **Figure S7.** MRT67307 and ruxolitinib attenuate interferon production and promote IAV replication. (a) A549 cells were not treated, pretreated with 1 μM MRT67307 for 30 min or pretreated with 4 μM ruxolitinib for 24 h. Next, A549 cells were infected with WSN (MOI = 1) for 6 h and then harvested. Cellular RNAs were extracted and measured by RT-qPCR. The levels of NP vRNA and IFNB mRNA were normalized by GAPDH mRNA. Values represent the mean ± SD of three independent experiments. (b) Expression of ISG15 and PKR mRNA were normalized by GAPDH mRNA from A549 cells that were treated as mentioned in (a). Values represent the mean ± SD of three independent experiments. Statistical comparisons between groups by one-way ANOVA with Bonferroni correction for multiple comparisons (a and b). *P < 0.05, **P < 0.01, and ***P < 0.001. (TIF 49 kb)

Additional file 8: **Figure S8.** Distribution of small RNAs in the extracellular and intracellular compartments. (a) Ratio of small RNAs in the extracellular and intracellular compartment in A549 cells without infection. The cell lysates and supernatants were collected from uninfected A549 cells and used for RNA extraction. Relative amounts of extracellular and intracellular small RNA were calculated. Values represent the mean ± SD of three independent experiments. (b) A549 cells were infected with influenza WSN (MOI = 1). Cell lysates and supernatants were collected at 24 h p.i. and the respective RNAs were extracted. Total RNA was extracted for RT-qPCR. Relative amounts of extracellular and intracellular small RNA were calculated. Values represent the mean ± SD of three independent experiments. (TIF 69 kb)

Additional file 9: **Figure S9.** GW4869 promotes IAV replication. A549 cells were treated with 10 μM GW4869 or DMSO and then infected with WSN at a MOI of 0.1 for 24 h. Cellular RNAs were extracted and measured by RT-qPCR. The levels of NP vRNA were normalized by GAPDH mRNA. Values represent the mean ± SD of three independent experiments. (TIF 11 kb)

Additional file 10: **Figure S10.** Protein quantification of exosome from donor cells. Protein concentrations of exosomes isolated from donor cells, which are transfected with control siRNA (Si_CTR) or hsa-miR-1975 inhibitor and then treated with mock or Poly(I:C), were measured by Bradford protein assay. Values represent the mean ± SD of three independent experiments. Statistical comparisons between groups by one-way ANOVA with Bonferroni correction for multiple comparisons. There is no statistical significance among these three groups. (TIF 18 kb)

Additional file 11: **Figure S11.** hsa-miR-1975 mimic or inhibitor does not influence cell survival. A549 cells were transfected with mock, control SiRNA (Si_CTR), hsa-miR-1975 mimic (mimic), hsa-miR-1975 inhibitor (inhibitor) for 48 h. For virus infected-group, A549 cells were infected with WSN at MOI = 1 for 24 h after transfection. MTS assay was used for the measurement of cell proliferation. Data presented with relative survail were compared to the mock treatemnet. Values represent the mean ± SD of three independent experiments. Statistical comparisons between groups by one-way ANOVA with Bonferroni correction for multiple comparisons. (TIF 179 kb)

Additional file 12: **Figure S12.** Expression of IFNB after transfection of hsa-miR-1975 mimic and scramble miR-1975. A549 cells were transfected with mock, control SiRNA (Si_CTR), scramble miR-1975 (scramble) or hsa-miR-1975 mimic (mimic). At 48 h post-transfection, A549 cells were infected with WSN (MOI = 1) for 6 h and then harvested. Cellular RNAs were extracted and measured by RT-qPCR. The levels of IFNB mRNA were normalized by GAPDH mRNA. Values represent the mean ± SD of three independent experiments. Statistical comparisons between groups by one-way ANOVA with Bonferroni correction for multiple comparisons. *P < 0.05. (TIF 122 kb)

## Data Availability

The datasets used and/or analyzed during the current study are available from the corresponding author on reasonable request.
