# Supplementary figures and images for: Exosome-delivered and Y RNA-derived small RNA suppresses influenza virus replication
Source: J Biomed Sci. 2019 Aug 15;26:58. doi: 10.1186/s12929-019-0553-6 (PMC6694579; doi:10.1186/s12929-019-0553-6)

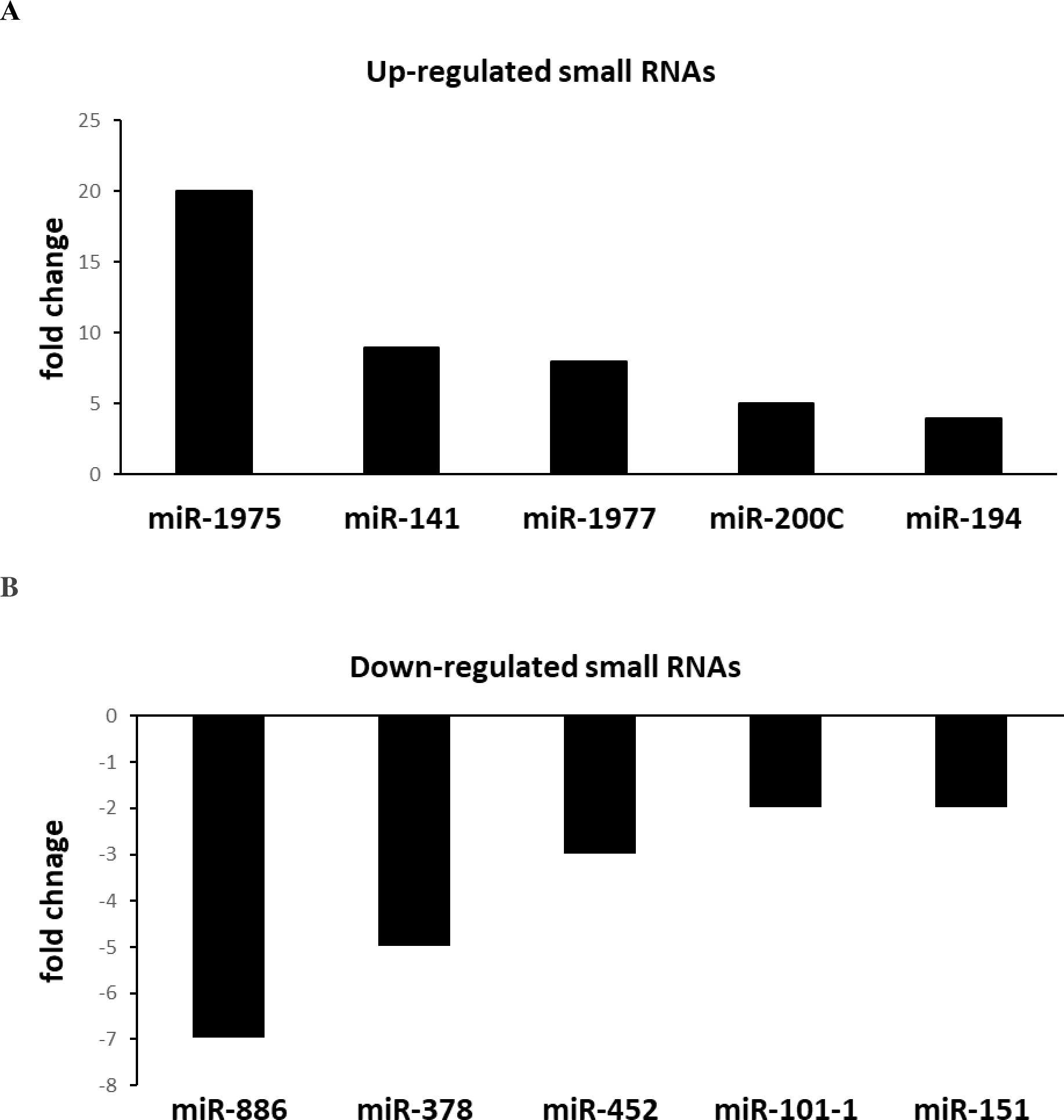

Supplement: Supplementary file 1 — Figure S1. Major up-regulated and down-regulated small RNAs in IAV-infected cells. (a) Top 5 up-regulated small RNAs. (b) Top 5 down-regulated small RNAs. Cellular small RNAs expression patterns in A549 cells infected with WSN were compared with those of uninfected A549 cells. Differentially expressed small RNAs with a fold change > 2 are described. (TIF 55 kb) [file 12929_2019_553_MOESM1_ESM.tif]

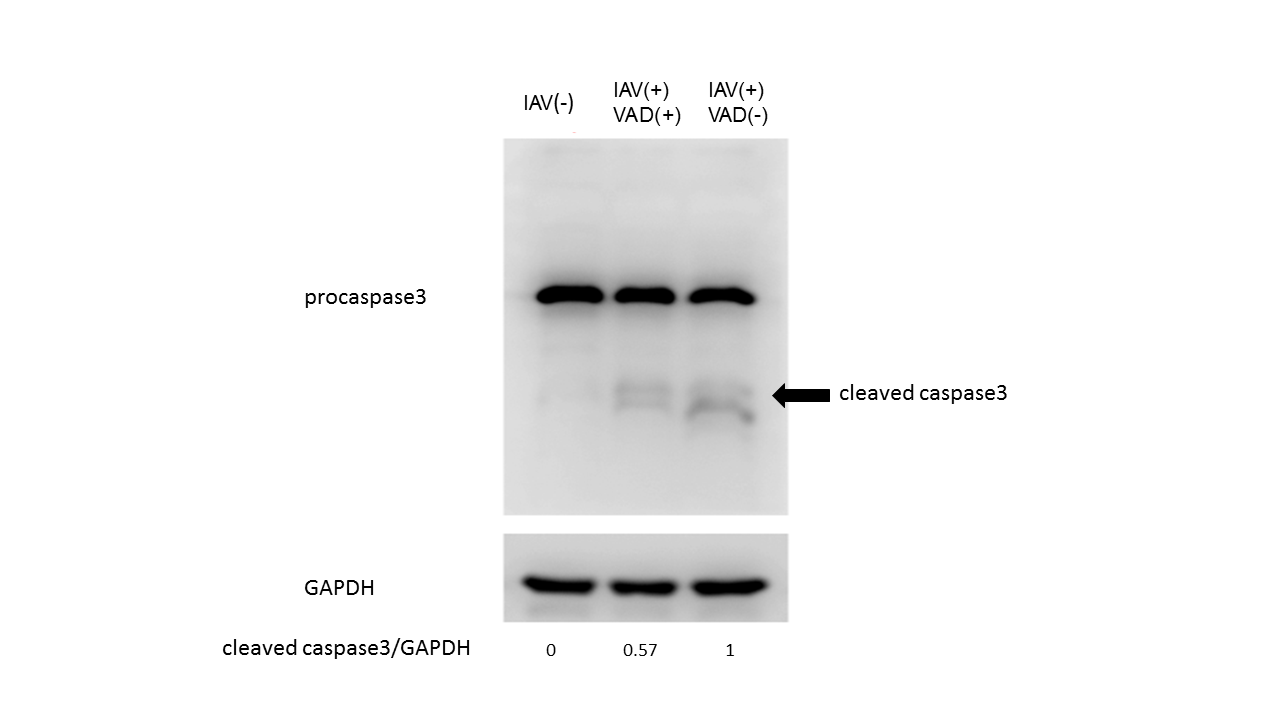

Supplement: Supplementary file 2 — Figure S2. Western Blot of Caspase3 and GAPDH after influenza infection and treated with VAD. A549 cells were treated with either mock or 20 μM VAD (a pan-caspase inhibitor) for 6 h and kept the treatment during virus infection and then infected with IAV (WSN) (MOI = 0.1) for 24 h or uninfected. WB of caspase3 and GAPDH was performed. The numbers below Western Blot are cleaved caspase3 protein ratio normalized by GAPDH. (TIF 177 kb) [file 12929_2019_553_MOESM2_ESM.tif]

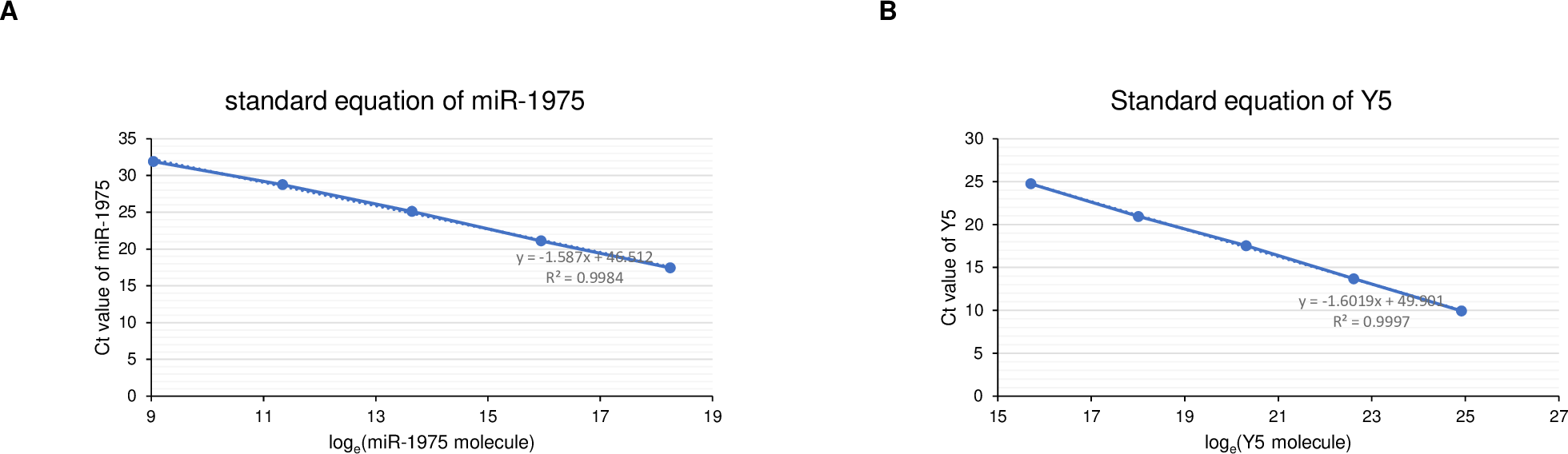

Supplement: Supplementary file 3 — Figure S3. (a) Standard equation of loge (number of miR-1975 molecules) and Ct value of miR-1975. (b) Standard equation of loge (number of Y5 molecules) and Ct value of Y5. (TIF 129 kb) [file 12929_2019_553_MOESM3_ESM.tif]

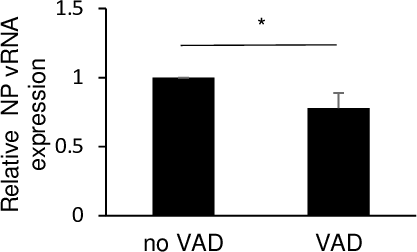

Supplement: Supplementary file 4 — Figure S4. Inhibition of apoptosis by VAD promotes IAV replication. A 549 cells were treated with either mock or VAD (a pan-caspase inhibitor) for 6 h. Relative expressions of NP viral RNA in A549 cells were compared. Values represent the mean ± SD of three independent experiments. Statistical comparisons between groups by Student’s t test. *P < 0.05. (TIF 7 kb) [file 12929_2019_553_MOESM4_ESM.tif]

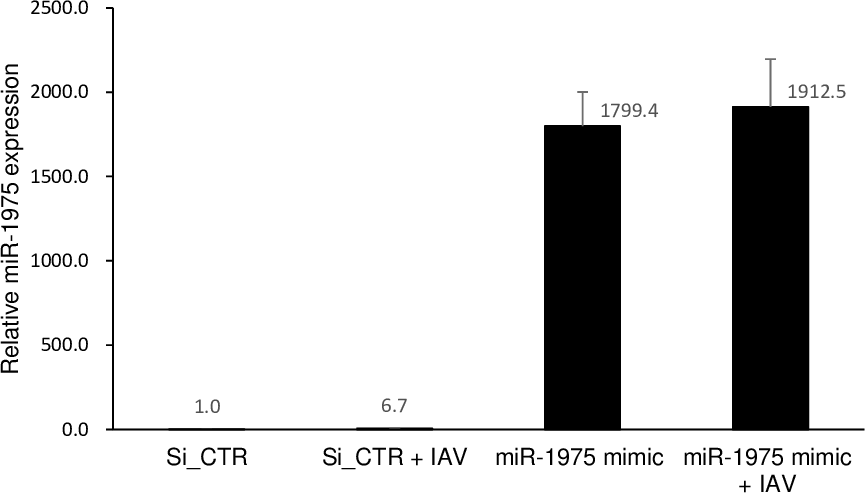

Supplement: Supplementary file 5 — Figure S5. Levels of endogenous and exogeneous miR-1975 in A549 cells after infected with WSN and miR-1975 mimic transfection. A549 cells were transfected with control SiRNA (Si_CTR) or miR-1975 mimic and then infected with WSN (MOI = 0.1) or not infected. At 24 h p.i., cells were harvested and processed for stem-loop RT-qPCR analysis of miR-1975. Values represent the mean ± SD of three independent experiments. (TIF 21 kb) [file 12929_2019_553_MOESM5_ESM.tif]

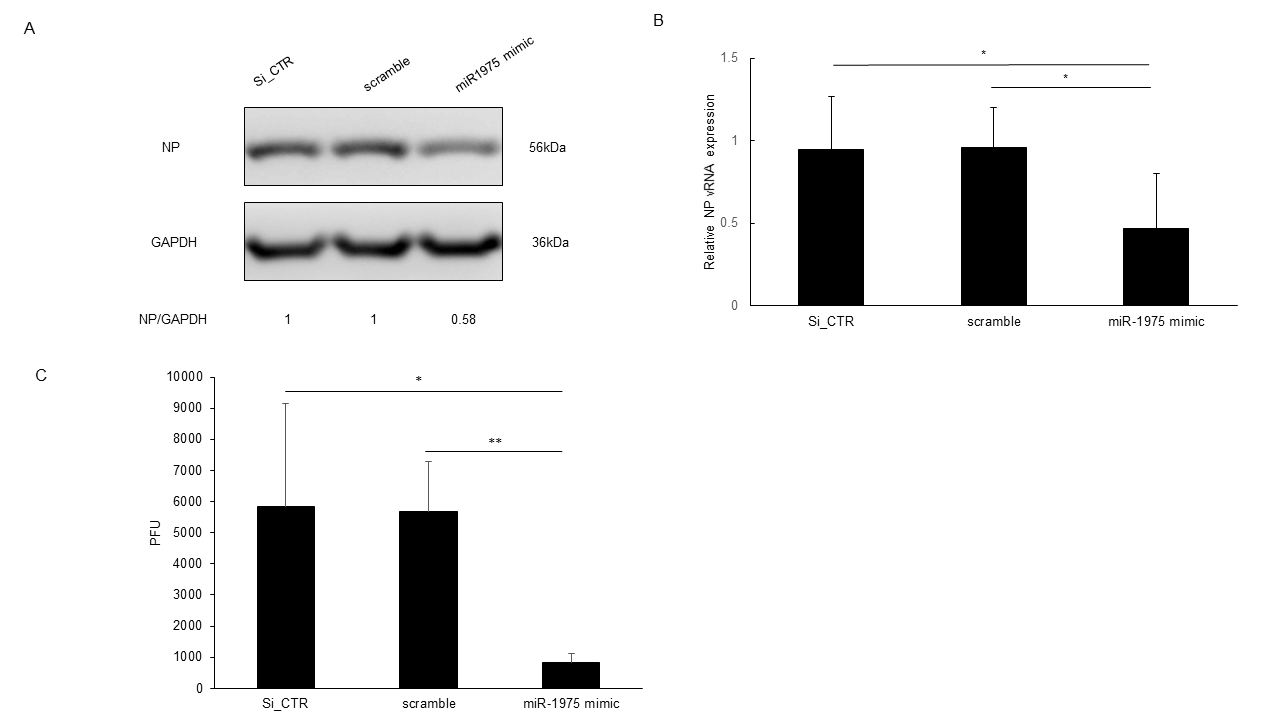

Supplement: Supplementary file 6 — Figure S6. Transfecting hsa-miR-1975 mimic reduces WSN replication. (a) and (b), A549 cells were transfected with control SiRNA (Si_CTR), scramble miR-1975 (scramble) and hsa-miR-1975 mimic and then infected with WSN at MOI of 1. At 6 h p.i., cells were harvested for immunoblotting of viral NP and cellular GAPDH proteins (a) or RT-qPCR of NP vRNA expression (b). The band intensities were quantified, and the relative NP/GAPDH ratios are shown below the blots (a). The relative NP expressions upon WSN infection were assessed by RT-qPCR. The expression levels were normalized by GAPDH (b). (c) A549 cells were infected with influenza WSN at an MOI of 0.1. The supernatants were collected at 24 h p.i and used for determining the viral titer by plaque assay in MDCK cells. Values represent the mean ± SD of three independent experiments. Statistical comparisons between groups by one-way ANOVA with Bonferroni correction for multiple comparisons. *P < 0.05 and **P < 0.01. (TIF 97 kb) [file 12929_2019_553_MOESM6_ESM.tif]

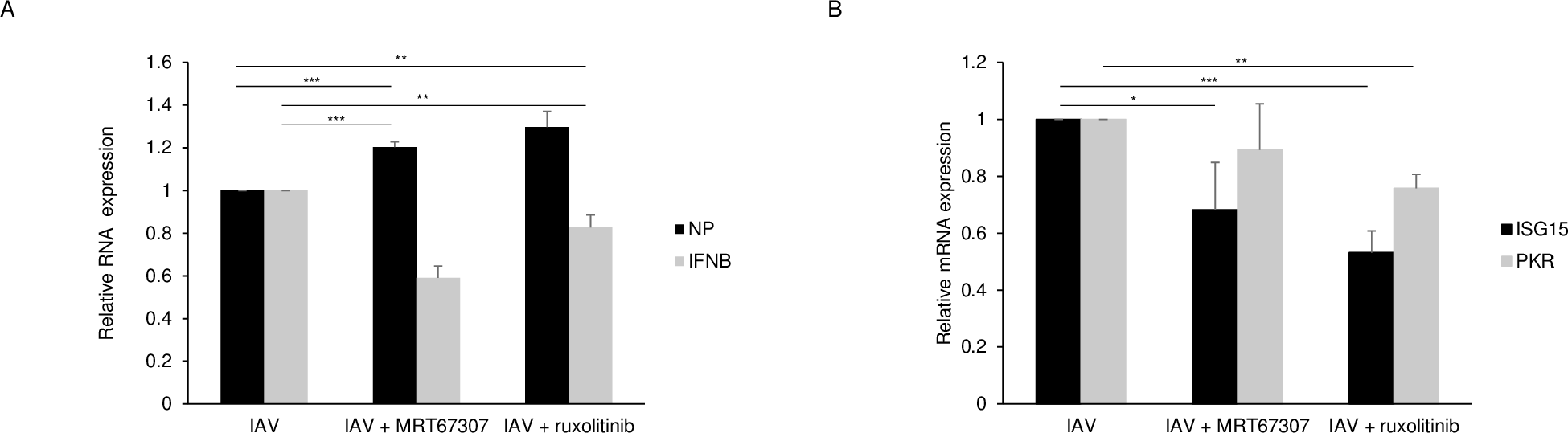

Supplement: Supplementary file 7 — Figure S7. MRT67307 and ruxolitinib attenuate interferon production and promote IAV replication. (a) A549 cells were not treated, pretreated with 1 μM MRT67307 for 30 min or pretreated with 4 μM ruxolitinib for 24 h. Next, A549 cells were infected with WSN (MOI = 1) for 6 h and then harvested. Cellular RNAs were extracted and measured by RT-qPCR. The levels of NP vRNA and IFNB mRNA were normalized by GAPDH mRNA. Values represent the mean ± SD of three independent experiments. (b) Expression of ISG15 and PKR mRNA were normalized by GAPDH mRNA from A549 cells that were treated as mentioned in (a). Values represent the mean ± SD of three independent experiments. Statistical comparisons between groups by one-way ANOVA with Bonferroni correction for multiple comparisons (a and b). *P < 0.05, **P < 0.01, and ***P < 0.001. (TIF 49 kb) [file 12929_2019_553_MOESM7_ESM.tif]

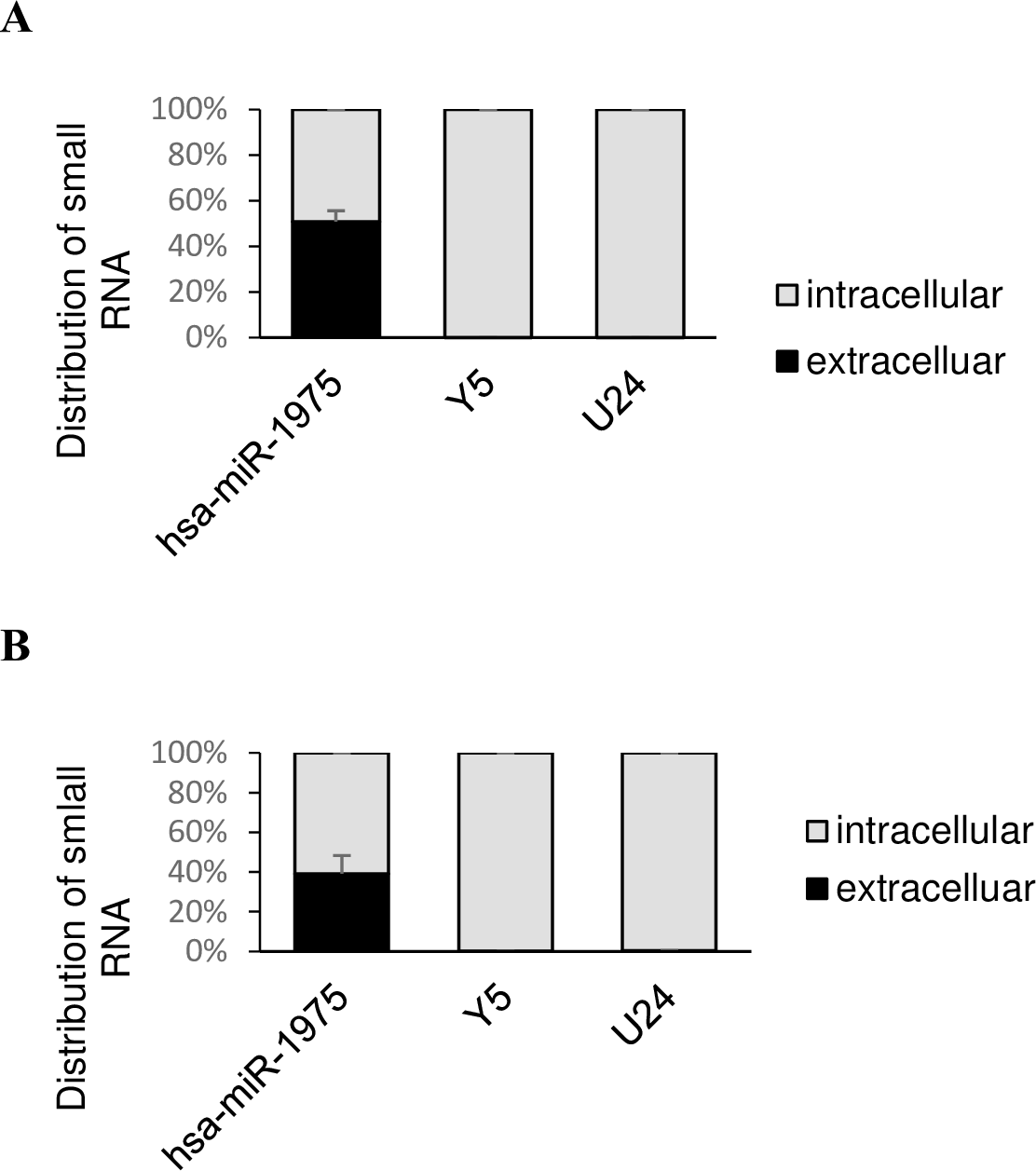

Supplement: Supplementary file 8 — Figure S8. Distribution of small RNAs in the extracellular and intracellular compartments. (a) Ratio of small RNAs in the extracellular and intracellular compartment in A549 cells without infection. The cell lysates and supernatants were collected from uninfected A549 cells and used for RNA extraction. Relative amounts of extracellular and intracellular small RNA were calculated. Values represent the mean ± SD of three independent experiments. (b) A549 cells were infected with influenza WSN (MOI = 1). Cell lysates and supernatants were collected at 24 h p.i. and the respective RNAs were extracted. Total RNA was extracted for RT-qPCR. Relative amounts of extracellular and intracellular small RNA were calculated. Values represent the mean ± SD of three independent experiments. (TIF 69 kb) [file 12929_2019_553_MOESM8_ESM.tif]

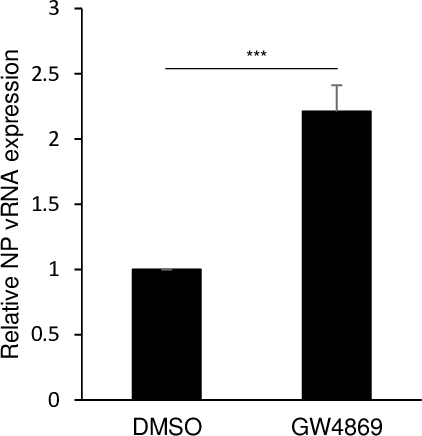

Supplement: Supplementary file 9 — Figure S9. GW4869 promotes IAV replication. A549 cells were treated with 10 μM GW4869 or DMSO and then infected with WSN at a MOI of 0.1 for 24 h. Cellular RNAs were extracted and measured by RT-qPCR. The levels of NP vRNA were normalized by GAPDH mRNA. Values represent the mean ± SD of three independent experiments. (TIF 11 kb) [file 12929_2019_553_MOESM9_ESM.tif]

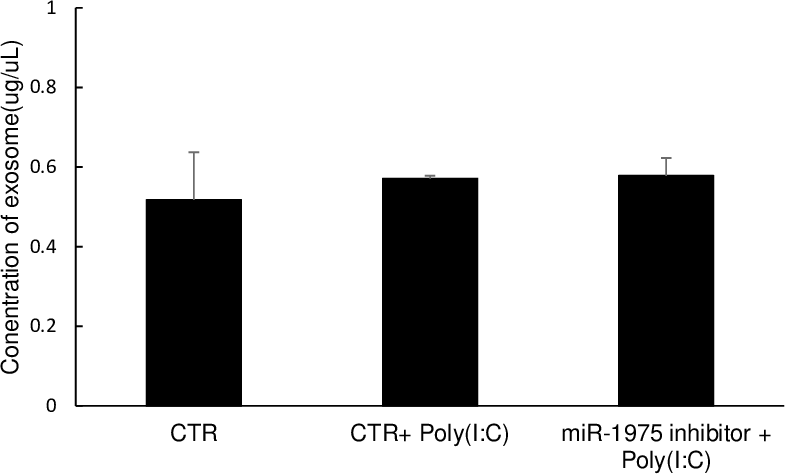

Supplement: Supplementary file 10 — Figure S10. Protein quantification of exosome from donor cells. Protein concentrations of exosomes isolated from donor cells, which are transfected with control siRNA (Si_CTR) or hsa-miR-1975 inhibitor and then treated with mock or Poly(I:C), were measured by Bradford protein assay. Values represent the mean ± SD of three independent experiments. Statistical comparisons between groups by one-way ANOVA with Bonferroni correction for multiple comparisons. There is no statistical significance among these three groups. (TIF 18 kb) [file 12929_2019_553_MOESM10_ESM.tif]

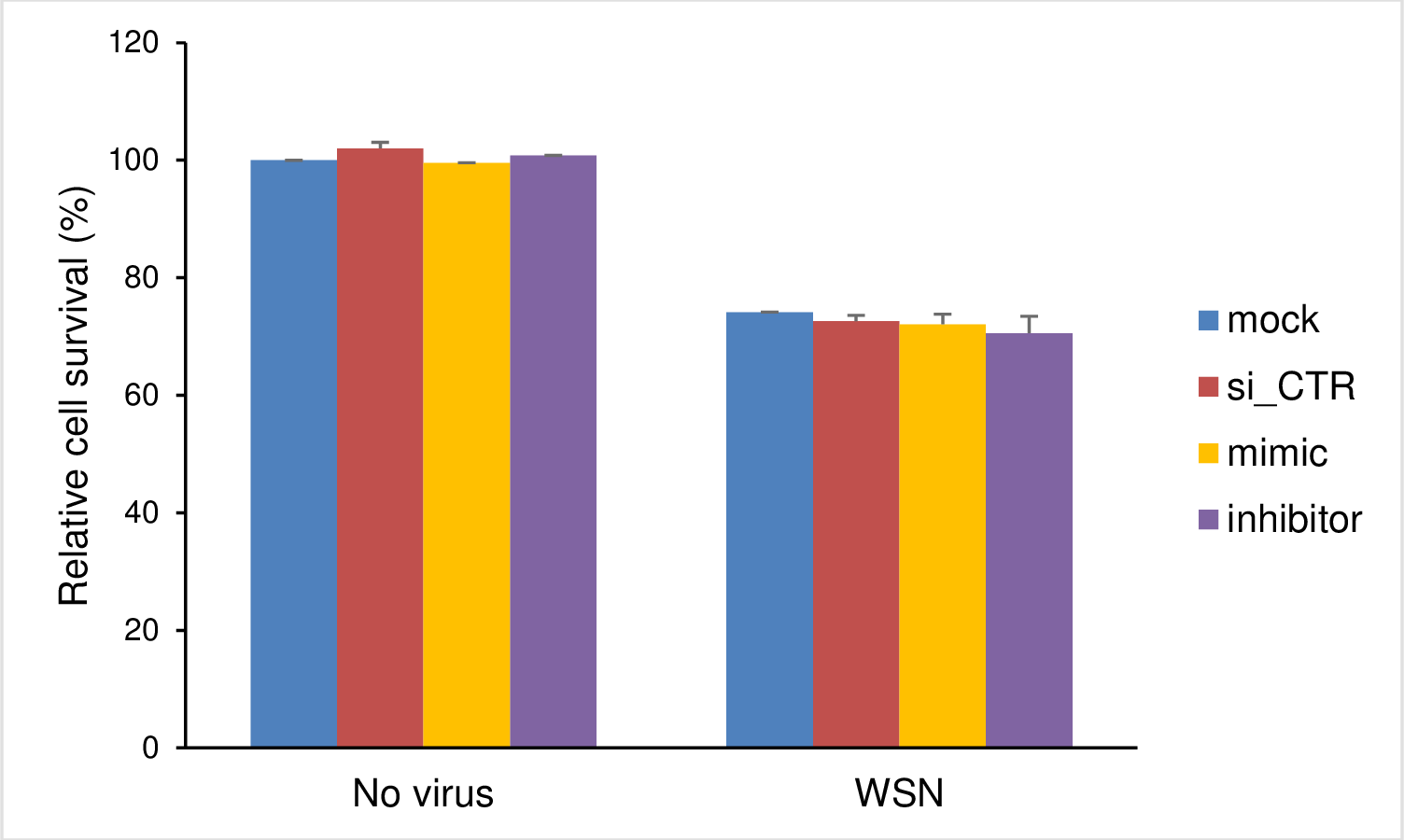

Supplement: Supplementary file 11 — Figure S11. hsa-miR-1975 mimic or inhibitor does not influence cell survival. A549 cells were transfected with mock, control SiRNA (Si_CTR), hsa-miR-1975 mimic (mimic), hsa-miR-1975 inhibitor (inhibitor) for 48 h. For virus infected-group, A549 cells were infected with WSN at MOI = 1 for 24 h after transfection. MTS assay was used for the measurement of cell proliferation. Data presented with relative survail were compared to the mock treatemnet. Values represent the mean ± SD of three independent experiments. Statistical comparisons between groups by one-way ANOVA with Bonferroni correction for multiple comparisons. (TIF 179 kb) [file 12929_2019_553_MOESM11_ESM.tif]

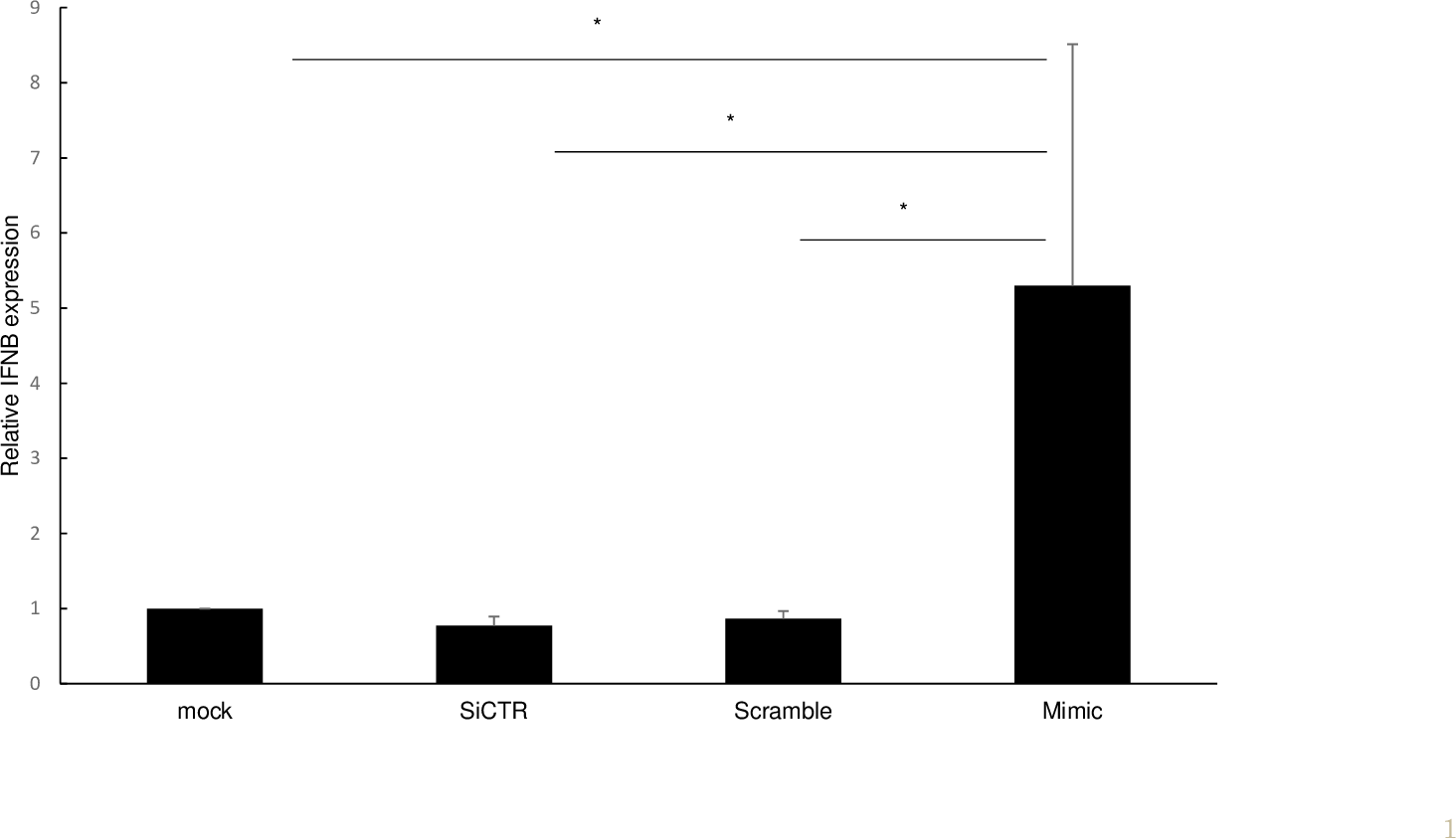

Supplement: Supplementary file 12 — Figure S12. Expression of IFNB after transfection of hsa-miR-1975 mimic and scramble miR-1975. A549 cells were transfected with mock, control SiRNA (Si_CTR), scramble miR-1975 (scramble) or hsa-miR-1975 mimic (mimic). At 48 h post-transfection, A549 cells were infected with WSN (MOI = 1) for 6 h and then harvested. Cellular RNAs were extracted and measured by RT-qPCR. The levels of IFNB mRNA were normalized by GAPDH mRNA. Values represent the mean ± SD of three independent experiments. Statistical comparisons between groups by one-way ANOVA with Bonferroni correction for multiple comparisons. *P < 0.05. (TIF 122 kb) [file 12929_2019_553_MOESM12_ESM.tif]
